# Global conformation of the Rag GTPase heterodimer governs eukaryotic amino acid sensing

**DOI:** 10.1073/pnas.2517050122

**Published:** 2025-10-15

**Authors:** Dylan D. Doxsey, Kuang Shen

**Affiliations:** ^a^Program in Molecular Medicine, University of Massachusetts Chan Medical School, Worcester, MA 01605; ^b^Department of Biochemistry and Molecular Biotechnology, University of Massachusetts Chan Medical School, Worcester, MA 01605

**Keywords:** mTORC1, Rag GTPase, single-molecule FRET, protein conformation, amino acid sensing

## Abstract

The Rag GTPase heterodimer is a central mediator of amino acid sensing in eukaryotic cells. When amino acids are abundant, it binds to the mechanistic target of rapamycin complex 1 to activate cellular programs for growth and proliferation. In its functional cycle, besides local conformational changes near the nucleotides that are commonly observed in monomeric signaling GTPases, the relative positioning of the two Rag subunits, i.e., the global conformation, is unique due to the heterodimeric architecture. Although various global conformations have been captured in static structural models, dynamic transitions between these conformations and their biological relevance remain unclear. Here, we visualize the global conformation of the Rag GTPase heterodimer using single-molecule Förster resonance energy transfer. By tracking the movement of individual protein molecules, we found that the two subunits explore a wide conformational space, which is strictly dictated by the bound nucleotides, regulators, and mutations. Additionally, we demonstrate that proper modulation of the global conformation is crucial for correctly interpreting amino acid signals. Our results defined a checkpoint of amino acid sensing in eukaryotic cells.

## Significance

The conformation of a protein—the way its amino acid chain folds into structures—determines how the protein functions and interacts with other molecules. This unique shape dictates the protein’s function, and changes in conformation may alter its biological function. This paper investigates how the Rag GTPase heterodimer, crucial for sensing amino acids, changes its overall shape (global conformation). Using single-molecule FRET, the study found that nucleotide binding, mutations, and interaction with mTORC1 alter these conformations. A conserved proline residue acts as a “hinge,” mediating these changes, and its mutation disrupts amino acid signaling. This work uncovers a checkpoint in amino acid sensing, emphasizing that the Rag GTPases’ global conformation is as vital as local nucleotide binding.

The mechanistic target of Rapamycin complex I (mTORC1) is a master regulator in eukaryotic cells that coordinates nutrient levels with cell growth and proliferation (1–4). mTORC1 is a serine/threonine protein kinase complex that upon activation, phosphorylates downstream effectors to boost mRNA translation, ribosomal biogenesis, and to inhibit autophagy. Activation of mTORC1 requires two steps mediated by two distinct GTPase units. First, in the presence of amino acids, the Rag GTPase heterodimer recruits mTORC1 to the lysosomal surface (5, 6). Second, when the PI3K-Akt-TSC signaling pathway is activated by growth factors, the lysosome-anchored Rheb GTPase binds to mTORC1 and triggers conformational changes to stimulate its kinase activity (7–10). These two branches ensure that eukaryotic cells only grow when both energy and nutrient resources are present.

The Rag GTPase heterodimer occupies a key position in the cellular response to amino acids. Upstream amino acid sensors such as Sestrin2 (11–14), CASTOR1 (15, 16), and SAMTOR (17, 18) directly bind to specific amino acids or their downstream metabolites, and transmit their abundance through a series of large protein complexes including GATOR1 (19, 20), GATOR2 (19), SLC38A9 (21, 22), and KICSTOR (23, 24). These regulators then manipulate the nucleotide loading configuration of the Rag GTPase heterodimer, enhancing or weakening its binding affinity for mTORC1, and thus controlling its subcellular localization. Unlike canonical monomeric signaling GTPases, each functional unit of the Rag GTPases contains two subunits, RagA or RagB tightly bound to RagC or RagD, with each subunit capable of binding guanine nucleotides (25, 26). When RagA/B binds guanosine triphosphate (GTP) and RagC/D binds guanosine diphosphate (GDP), the Rag GTPase heterodimer is considered activated and strongly interacts with mTORC1, facilitating its recruitment (5, 6). In contrast, when RagA/B binds GDP and RagC/D binds GTP, the Rag GTPase heterodimer becomes inactivated and the affinity to mTORC1 weakens. This coordinated change of nucleotide loading configurations allows for additional layers of regulation in the amino acid sensing process.

Nucleotide loading on the two Rag subunits is strictly regulated. For each Rag subunit, an N-terminal nucleotide binding domain (NBD) binds guanine nucleotides, and a C-terminal roadblock domain (CRD) mediates heterodimerization (25, 26). Biochemical studies have identified an internal “locking mechanism” that coordinates nucleotide loading on the two subunits (27). When one of the two subunits binds GTP, it takes a dominant role and allosterically controls the behavior of the other subunit by greatly reducing the affinity of a second GTP binding. In addition, the subunit with the prebound GTP stimulates hydrolysis of the later-bound GTP if accidental binding happens. These mechanisms ensure that only one subunit is loaded with GTP and a defined signal is transmitted to mTORC1 and other potential effectors.

The conformation of the Rag GTPase heterodimer varies dramatically across different structural models. Because of the heterodimerized architecture, both local and global conformational changes have been observed. The local conformational change happens near the nucleotide binding pocket and is strongly coupled to nucleotide identity. Upon GTP binding, Switch I swings to the top of the nucleotide binding pocket, forming a lid (28). When a GDP molecule binds, Switch I relaxes and extends toward the CRD (29). This feature is common in Arf-family GTPases and has been observed in canonical monomeric signaling GTPases (30, 31). In contrast, the global conformational change is unique to the Rag GTPase heterodimer and depends on the bound nucleotides, mutations, and the binding partners. While the two CRDs in the heterodimer bind each other strongly and statically, the relative positioning of the two NBDs, i.e., the global conformation, varies, suggesting a potentially dynamic nature (*SI Appendix*, Fig. S1*A*).

Structural studies have so far unveiled three general classes of global conformations. In the first class, where RagA binds GTP and RagC binds GDP, the two NBDs contact each other to adopt a closed conformation (29). This is considered the active state. The second class is the open conformation where the two NBDs rotate away from one another. Several conditions can lead to this conformation such as dual-GTP loading (28), as well as Raptor (32, 33) and GATOR1 binding (34, 35). The third class is a wide-open conformation (36–38), which has only been observed when the Rag GTPase binds FLCN–FNIP2, a GTPase-activating protein for RagC. Here, the NBD of RagA is pushed outward, opening up the interdomain space to allow for FLCN–FNIP2 binding. As structural models generally provide static snapshots, we do not know the dynamic transitions between conformations, nor their roles in mTORC1 signaling in cells.

Based on the structural information and the biochemical evidence, we hypothesized that the global conformation of the Rag GTPase heterodimer and the dynamics may be vital for its function. In this study, we used single-molecule Förster resonance energy transfer (smFRET) to visualize the conformations of the Rag GTPases. We identified a critical proline residue between NBD and CRD that governs the global conformation and dynamics. We also used cell biological tools to show that proper control of the global conformation is essential for faithful transmission of amino acid signals. Our results defined a checkpoint of the amino acid sensing process in eukaryotic cells.

## Results

### Establishment of the smFRET System.

To explore the conformational space of the Rag GTPase heterodimer using smFRET, we need to fluorescently label the two subunits with donor and acceptor dyes. Wild-type Rag GTPases contain multiple, surface-exposed cysteine residues, preventing site-specific labeling at desired positions (*SI Appendix*, Fig. S1*B*, lanes 1 and 4). Therefore, we engineered a “cyslite” version of the Rag GTPases, in which the surface-exposed cysteine residues were mutated (see Methods). This construct provided a clean background (*SI Appendix*, Fig. S1*B*, lanes 2 and 5), in which thiol-reactive fluorescent dyes failed to link to either RagA or RagC. Based on this construct, we can then introduce a single cysteine residue at desired positions to allow for site-specific labeling using maleimide-conjugated fluorescent dyes (*SI Appendix*, Fig. S1*B*, lanes 3 and 6). As a proof-of-principle experiment, we introduced a cysteine residue on RagA at amino acid position 80 and on RagC at amino acid position 190. We were able to detect covalent linking of BODIPY-FL to the corresponding subunit (*SI Appendix*, Fig. S1*C*).

We validated the functional integrity of the cyslite construct in both biochemical and cell biological contexts. First, using a crosslinking assay, we measured the binding affinity of nucleotides to wild-type and cyslite Rag GTPases (27). We incubated the Rag GTPases with radioactively labeled nucleotides, and then applied ultraviolet (UV) light to the reaction mixture to induce zero-distance crosslinking. By resolving the two subunits using SDS-PAGE, we differentiated nucleotides crosslinked, and thus bound, to RagA from those to RagC. We found that cyslite Rag GTPases show similar binding affinity of GTP on both subunits compared with wild-type Rag GTPases (*SI Appendix*, Fig. S1 *D* and *E*), suggesting the mutations did not cause nucleotide binding defects.

Using a hydrolysis assay, we measured the intrinsic GTP hydrolysis rate of the Rag GTPases (27). Here, we incubated the Rag GTPases with radioactively labeled GTP and monitored the percentage of hydrolyzed substrate over time. By tracing the reaction process, we found that cyslite Rag heterodimer hydrolyzes GTP at a similar rate to wild-type Rag heterodimer (*SI Appendix*, Fig. S1*F*), suggesting the mutations we introduced do not disrupt the catalytic center of the GTPase.

We checked the interaction of the Rag GTPases with their upstream regulators by performing a stimulated hydrolysis assay (27). GATOR1, a GTPase-activating protein (GAP) for RagA, was added to GTP-loaded Rag GTPases to stimulate the hydrolysis reaction. Again, we observed a similar amplitude of stimulation for cyslite and wild-type Rag GTPases (*SI Appendix*, Fig. S1*G*), suggesting the mutations do not disrupt the interaction between the Rag GTPases and GATOR1.

Finally, we introduced cyslite Rag GTPases into cells and evaluated their ability to mediate amino acid signals in vivo. Here, we used HEK-293 T cell lines in which endogenous Rag GTPases were knocked out using CRISPR-Cas9 (24) (sgRagA/B or sgRagC/D). Compared to wild-type HEK-293Ts (*SI Appendix*, Fig. S1 *H* and *I*, lanes 1 and 2), sgRagA/B and sgRagC/D cell lines became unresponsive to nutrients in the culturing media, as reflected by the low and unchanged phosphorylation of S6K1 on the Thr389 residue in the absence or presence of amino acids (*SI Appendix*, Fig. S1 *H* and *I*, lanes 3 and 4). While reexpression of wild-type RagA and RagC restores the sensitivity of sgRagA/B and sgRagC/D cell lines to amino acids (*SI Appendix*, Fig. S1 *H* and *I*, lanes 5 and 6), cyslite RagA and RagC are also capable of doing so (*SI Appendix*, Fig. S1 *H* and *I*, lanes 7 and 8). Taken together, our results support the functional integrity of cyslite Rag GTPases in vitro and in vivo, so we consider it equivalent to wild-type in this manuscript.

**Fig. 1.**
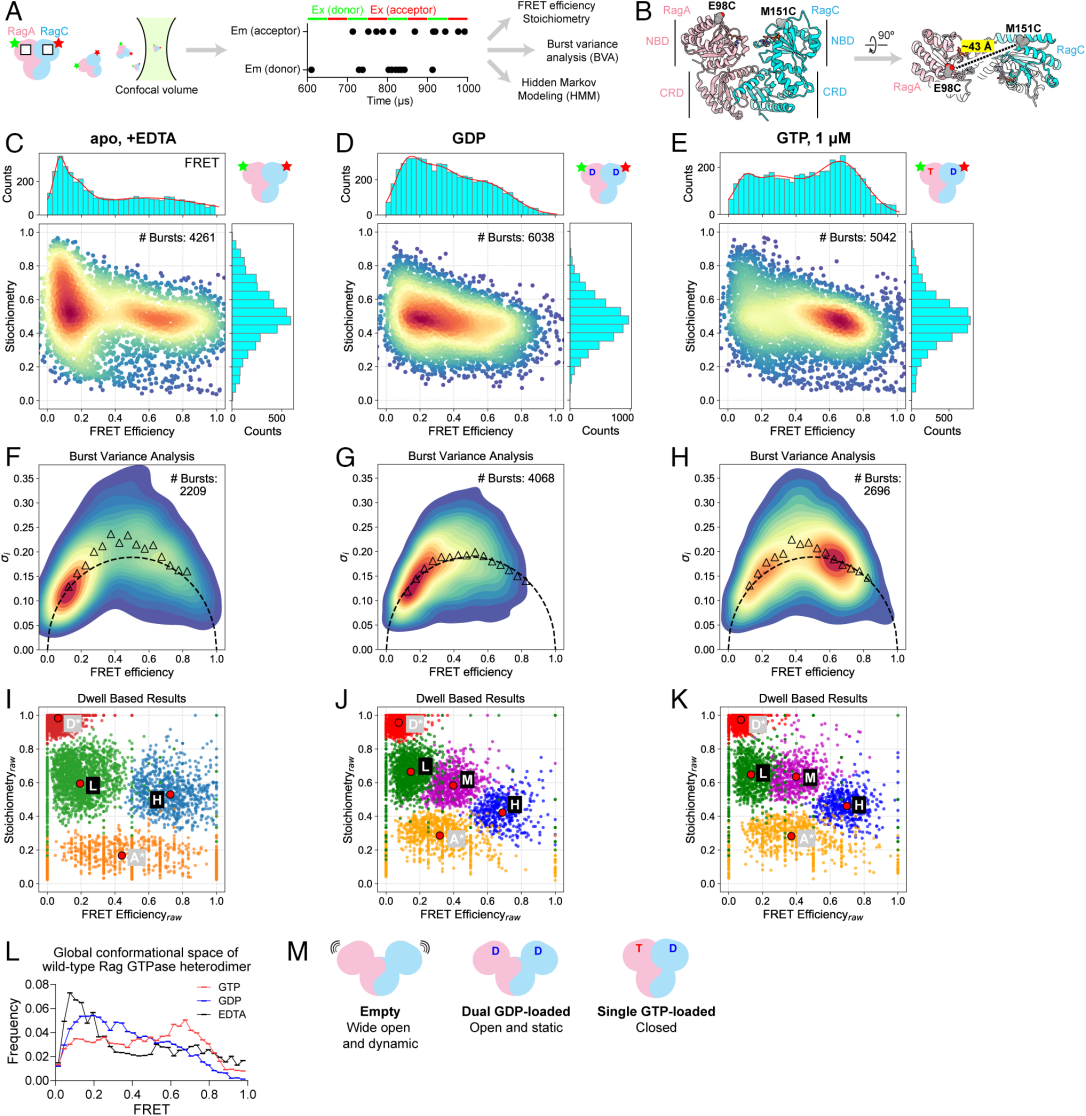
Global conformation of the Rag GTPases is regulated by the nucleotide loading configurations. (*A*) Alternating-laser excitation-based single-molecule setup to detect the global conformation of the Rag GTPases. White boxes represent nucleotide binding pockets. (*B*) Fluorescent labeling scheme for the Rag GTPases. RagA is labeled at residue 98, and RagC is labeled at residue 151. (*C*–*E*) FRET efficiency-stoichiometry (*E*-*S*) diagrams of the Rag GTPases in its apo (*C*), GDP-bound (*D*), and single GTP-bound (*E*) nucleotide loading configuration. (*F*–*H*) Burst variance analyses (BVA) of the Rag GTPases in its apo (*F*), GDP-bound (*G*), and single GTP-bound (*H*) nucleotide loading configuration. Experimental values are shown in triangles. The static limit is shown as a dashed semicircle. (*I*–*K*) Hidden Markov modeling (HMM) of the bursts reveals hidden states in apo (*I*), GDP-bound (*J*), and single GTP-bound (*K*) nucleotide loading configuration. (*L*) Overlaying of the FRET histograms of the Rag GTPases in apo, GDP, and single GTP-loaded state. (*M*) Scheme of the global conformation of the Rag GTPases.

With the cyslite Rag GTPases in hand, we designed a series of double cysteine mutants, one on each subunit at different positions. We then statistically labeled these mutants^*^ with donor (Cy3) and acceptor (Cy5) dyes and carried out alternating laser excitation (ALEX)-based single-molecule experiments to measure the FRET efficiency (39) (Fig. 1*A*). Here, protein samples are diluted to sub-nanomolar concentration and illuminated by a confocal laser beam. Statistically, only one protein molecule will diffuse through the confocal volume at a certain time. During this time period, usually several milliseconds, the fluorescent dyes attached are excited by alternating donor and acceptor lasers. Emitted donor and acceptor photons can then be split and recorded with Avalanche photodiodes (APDs) and subjected to analyses (Fig. 1*A*). Most notably, two parameters can be directly calculated for each burst: the FRET efficiency (*E*) and donor-to-acceptor stoichiometry (*S*). In particular, if a protein molecule is dual labeled with two donor dyes, acceptor fluorescence will not be detected during the period when the acceptor laser excites (*S*~1, *SI Appendix*, Fig. S1 *J*, *Top Left* corner). Alternatively, if a protein molecule is dual labeled with two acceptor dyes, donor fluorescence will not be detected during the period when the donor laser excites (*S*~0, *SI Appendix*, Fig. S1 *J*, *Bottom Right* corner). By excluding the two unwanted populations above, ALEX can selectively observe protein molecules that are labeled with both donor and acceptor dyes (*S*~0.5, *SI Appendix*, Fig. S1 *J*, *Middle*). Taking advantage of this feature, we can reliably explore the conformational space of the Rag GTPase heterodimer on the single-molecule level.

### ALEX-Based smFRET to Monitor the Conformational Space of the Rag GTPase Heterodimer.

We first labeled RagA and RagC on their nucleotide binding domains (NBDs), at amino acid positions 98 and 151, respectively (Fig. 1*B*). Based on previously resolved structural models, the distance between the α-carbons of the two residues ranges from 43 to 58 Å, providing a sensitive readout of the global conformation (*SI Appendix*, Fig. S1*A*). In its apo state, where we stripped the bound nucleotides with EDTA, we acquired 4,261 bursts from dual-labeled protein molecules that passed through the confocal volume (Fig. 1 *C*, *E*–*S* plot, *Bottom* panel). When we calculated the FRET values of individual bursts and plotted their distribution, we observed two major populations (Fig. 1 *C*, *Top* panel). First, a dominant low-FRET population was found at around the FRET efficiency of ~0.13. This FRET value corresponds to a distance of ~74 Å between the donor- and acceptor-dyes^†^, significantly longer than the distances measured from static structural models, suggesting the NBDs of the two Rag subunits drift apart from one another into a wide-open conformation in the absence of any bound nucleotides. Second, we identified a population of molecules with FRET values ranging from 0.4 to 0.85, suggesting that the two NBDs come close to one another occasionally. Notably, the distribution of the high-FRET population is much broader than that of the low-FRET population, likely reflecting the heterogeneity of conformations within.^‡^

To probe the conformational dynamics, we carried out burst-variance analysis (40) (BVA, Fig. 1*F*). Here, each burst is divided into multiple subbursts, and their FRET values are calculated individually (*E*_sub_s). If a protein molecule only adopts a single (static) conformation when passing through the confocal volume, the SD (σ) of *E*_sub_s should follow that of a binomial distribution (40) (Fig. 1*F*, dashed semicircle). In contrast, if a protein molecule quickly transitions between multiple conformations, the SD of *E*_sub_s should be greater than the static values—this is indeed the case for the Rag GTPase heterodimer in its apo state. When we calculated the SD of *E*_sub_s (Fig. 1*F*, triangles), we found a significant deviation from the static standards, especially for the high-FRET population (Fig. 1*F*, FRET > 0.4). This result suggests that the Rag GTPases go through rapid conformational changes when no nucleotide is loaded, and the high-FRET state is likely transiently explored without being stably occupied.

To quantitatively determine the transition kinetics, we applied multiparameter, photon-by-photon hidden Markov modeling to our system (41, 42) (mpH^2^MM, HMM for short, Fig. 1*I*). Donor and acceptor photon streams were directly fed into the HMM algorithm, and the (hidden) FRET states and transition kinetics can be extracted. For the apo-Rag GTPase dataset, we applied a four- (Fig. 1*I*) or five-state model (*SI Appendix*, Fig. S2*B*) to describe the behavior based on integrated complete likelihood criterion (ICL, *SI Appendix*, Fig. S2*A*). Besides the donor-only (Fig. 1*I*, D*, red) and acceptor-only state (Fig. 1*I*, A*, orange), the four-state model reveals two real FRET states (Fig. 1*I* and *SI Appendix*, Fig. S2*C*): a low-FRET state (Fig. 1*I*, **L**, green) with a FRET value of 0.19 and a high-FRET state (Fig. 1*I*, **H**, blue) with a FRET value of 0.73. The transition rate from **L** state to **H** state is 50 ± 4 s^−1^, while the transition rate from **H** state to **L** state is faster, at 202 ± 14 s^−1^. These results are consistent with the FRET histogram (cf. Fig. 1*C*) and BVA (cf. Fig. 1*F*), suggesting that the two NBDs within the Rag GTPase heterodimer mostly reside distant from one another in the absence of any bound nucleotides, while only transiently exploring a more closed conformation.

### A Single GTP Binding Induces Closure of the Rag GTPase Heterodimer.

GTPases switch conformations when they are loaded with different nucleotides to carry out various functions. Because the Rag GTPase heterodimer has four nucleotide loading configurations, we first probed its global conformation when both subunits bind GDP by incubating prestripped Rag GTPases with a saturating concentration of GDP (100 μM). When we plotted the FRET efficiency of 6,038 molecules, we found a distinct pattern. Besides a low-FRET population, we identified a more defined, medium-FRET population with the FRET value of ~0.35, and a lowly populated high-FRET population with the FRET value of ~0.6 on the histogram (Fig. 1 *D*, *Top* panel). The distances between dyes in medium- and high-FRET populations correspond to 61 and 51 Å, respectively, which are consistent with the open and closed conformations of the NBDs observed in structural models (28, 29). Moreover, the conformational dynamics of the dual-GDP loaded state also differs significantly from that of the apo state. BVA shows a rather static behavior, in which the SD of *E*_sub_s closely follows the static limit for all FRET values (Fig. 1*G*, triangles vs. dashed semicircle). Therefore, with two GDP molecules loaded, the NBDs within the Rag GTPase heterodimer remain steadily open, but start to probe a closed conformation.

To further test the result from the FRET histogram, we applied HMM to the dual-GDP dataset. Three FRET states can be readily resolved by a five-state model (Fig. 1*J* and *SI Appendix*, Fig. S2 *D* and *E*). Besides the **L** and **H** states that have been revealed in the apo condition, we detected an intermediary state (Fig. 1*J*, **M**, magenta) that corresponds to the medium-FRET population from frequency-based analyses (Fig. 1*D*). Interestingly, this **M** state is an on-pathway intermediate between **L** and **H**, as no direct transition between **L** and **H** can be identified by the *Viterbi* algorithm. Taken together, these results further corroborate our conclusion on the conformational space of dual-GDP bound Rag heterodimer.

In order to determine the effects of GTP loading on the heterodimer, we took advantage of the stark difference between the binding constants (*K*_d_) of the first- and second-bound GTP, as the empty heterodimer binds the first GTP molecule with a low-nanomolar affinity, while the remaining binding pocket has a *K*_d_ of ~2 μM (27). When we incubated the Rag GTPase heterodimer with 1 μM GTP and excess GDP, we observed a striking shift of the FRET distribution (Fig. 1*E*). Here, single-GTP loading leads to a predominant high-FRET population, together with diminished low- and medium-FRET populations. Compared with the dual-GDP loaded configuration (Fig. 1*H*), the dramatic shift suggests a major compaction of the global conformation of the Rag GTPase heterodimer: The two NBDs come close to one another, forming contacts that “locks” the functional unit. This high-FRET population is stable in dynamics, as BVA shows consistency between the measured values (Fig. 1*H*, triangles) and the static limit (Fig. 1*H*, dashed semicircle) at around the FRET efficiency of 0.6. In contrast, we detected dynamic behaviors of the medium-FRET population (Fig. 1*H*, FRET ~ 0.4), suggesting that the open conformation is relatively unstable under single-GTP loaded configuration. We further evaluated these findings using HMM (Fig. 1*K* and *SI Appendix*, Fig. S2 *F* and *G*), and confirmed the augmented high-FRET state.

To independently test the nucleotide-induced global conformational change, we performed the following control experiments. First, we designed two additional double-cysteine mutants on the NBDs, RagA(T75C)-RagC(E152C) (*SI Appendix*, Fig. S3 *A–E*), and RagA(E100C)-RagC(E152C) (*SI Appendix*, Fig. S3 *F*–*J*) and repeated the measurements above. Consistent with our prediction, both constructs show a wide-open conformation in their apo state, a low-FRET dominated conformation under dual-GDP loaded condition, and a high-FRET dominated conformation under single-GTP loaded condition. Second, as a negative control, we designed a construct in which the two dyes are placed on the C-terminal roadblock domains (CRDs) (*SI Appendix*, Fig. S3*K*). As CRDs mediate the heterodimerization of the Rag GTPases and remain steady regardless of the nucleotide loading configuration, we expect a constant FRET population under different conditions. This is indeed the case—a constant FRET distribution was observed across the board (*SI Appendix*, Fig. S3 *L–O*), suggesting no detectable movement of CRDs occurs when the NBDs go through the conformational changes.

Overall, the results above demonstrated that nucleotides modulate the global conformation of the Rag GTPase heterodimer (Fig. 1 *L* and *M*), in which a single GTP binding securely locks the Rag GTPases into a compact and stable conformation.

### Dual-GTP Loading Induces Crosstalk and Dynamics Within the Rag GTPase Heterodimer.

**Fig. 2.**
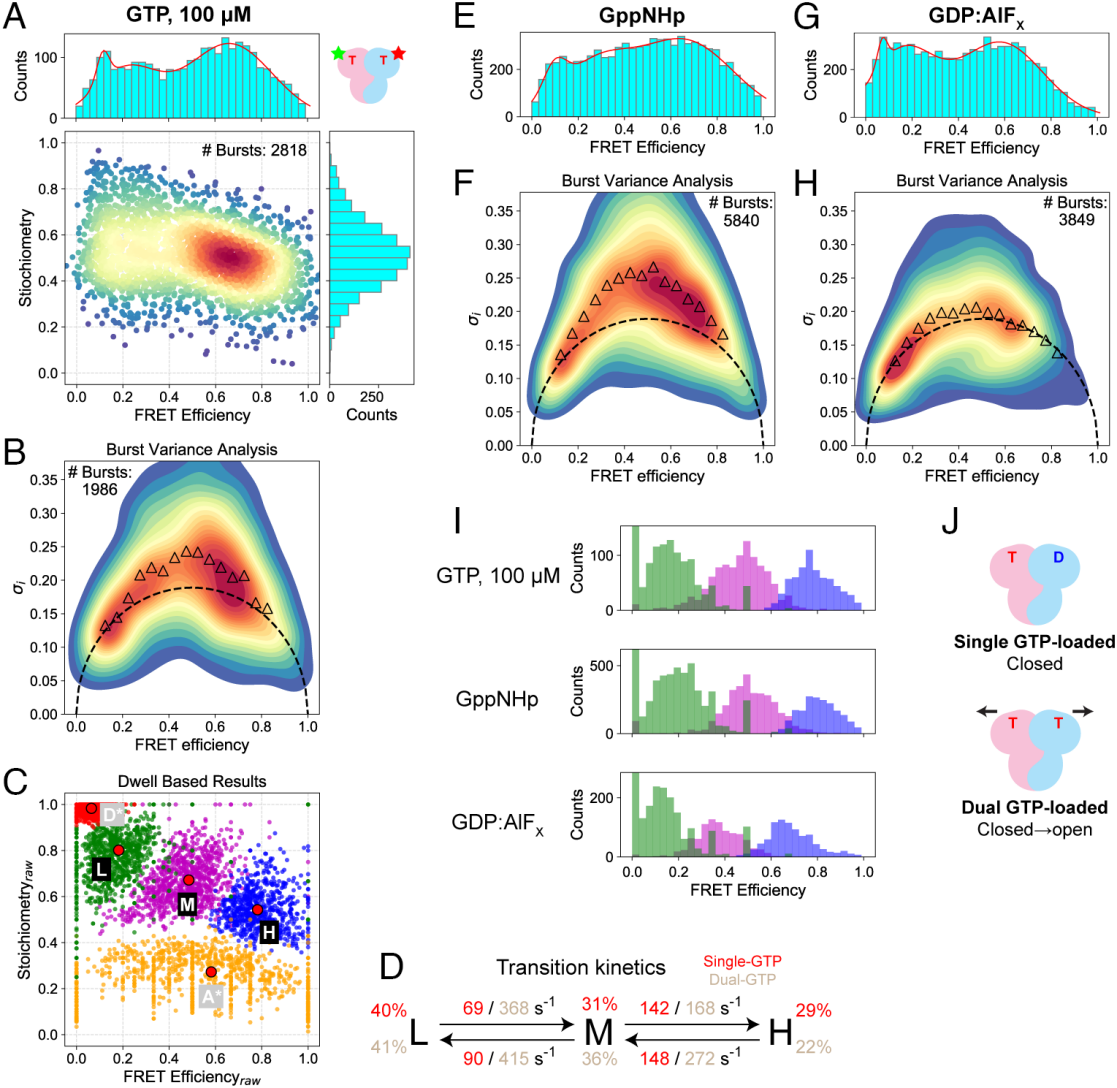
Dual GTP-loading induces crosstalk between the Rag subunits and opens up the heterodimer. (*A*) *E*-*S* diagram of the Rag GTPases in dual GTP-loaded state. (*B*) BVA of the Rag GTPases in dual GTP-loaded state. Experimental values (triangles) significantly deviate from the static limit (dashed semicircle). (*C*) HMM of the dual GTP-loaded Rag GTPases. (*D*) HMM reveals the transition rates. Data for single GTP-loaded Rag GTPases are shown in red, and those for dual GTP-loaded Rag GTPases are in brown. (*E*) *E*-*S* diagram of the Rag GTPases in dual GppNHp-loaded configuration. (*F*) BVA of the Rag GTPases in dual GppNHp-loaded configuration. (*G*) *E*-*S* diagram of the Rag GTPases in dual GDP:AlF_X_-loaded configuration. (*H*) BVA of the Rag GTPases in dual GDP:AlF_X_-loaded configuration. (*I*) Comparison of FRET states when the Rag GTPases are loaded with different nucleotides. (*J*) Dual GTP-loading on the Rag GTPases induces intersubunit crosstalk to separate the two NBDs apart.

A key feature of the Rag GTPase heterodimer is its unique nucleotide loading configuration, in which the strongest signaling effects in cells are observed when the two subunits bind to “opposite” nucleotides (GTP vs. GDP). Biochemical evidence suggests that when two GTP molecules bind simultaneously, intersubunit crosstalk initiates to resolve this configuration by specifically stimulating the hydrolysis of the later-bound GTP (27). To visualize this process, we performed smFRET measurements with a saturating concentration of GTP (100 µM), in which both subunits were forced to bind GTP. Compared with the single-GTP loaded condition, the high-FRET population under dual-GTP loaded condition declines, while the medium-FRET population increases (Fig. 2*A*). More strikingly, we detected dramatic changes in the dynamics within the Rag heterodimer: The two NBDs show much greater in-burst variation as BVA reveals significant deviations from the static standard (Fig. 2*B*). This behavior is not obvious in single-GTP loaded condition (cf. Fig. 1*H*), suggesting here the two subunits are in the process of resolving the dual-GTP loaded configuration. To further confirm this result, we carried out HMM to quantify the transition kinetics (Fig. 2*C*). While the FRET values of the three states are similar in both single- and dual-GTP loaded conditions, the transition kinetics unveil a sharp difference (Fig. 2*D*). Transitions between **L** and **M** states are much more active in dual-GTP loaded condition, while **H** to **M** transition is twice as fast than that in single-GTP loaded condition. The overall effect here results in a decrease of the closed conformation (**H** state) and an overpopulation of the open conformation (**M** state), further corroborating the FRET histogram observed from frequency-based analyses.

To pinpoint the precise moment when intersubunit crosstalk starts, we employed two nonhydrolyzable GTP analogs: GppNHp, a ground state mimetic (43), and GDP:AlF_X_, a transition state mimetic (44). When we incorporated these two analogs on the Rag GTPases, we observed very different behaviors. In the case of GppNHp, the medium-FRET population is more pronounced (Fig. 2*E*), suggesting that the Rag heterodimer is more populated in the open conformation. Moreover, BVA reveals active in-burst variations (Fig. 2*F*). As GppNHp mimics the “bound” state of GTP, these results suggest that as soon as the second GTP molecule binds, the Rag GTPase heterodimer is driven out of the stable, closed conformation, and dynamic intersubunit crosstalk initiates to resolve this dual-GTP bound configuration. In contrast, the loading of GDP:AlF_X_ triggers increases in both the medium- and low-FRET populations (Fig. 2*G*), while stabilizing these conformations (Fig. 2*H*). As GDP:AlF_X_ mimics the stage when GTP is about to be hydrolyzed, this behavior suggests that the intersubunit crosstalk likely concludes when the bound GTP enters the transition state and starts to hydrolyze, and the static, open conformation is the consensus made by the Rag subunits. HMM analyses further confirm these results (Fig. 2*I* and *SI Appendix*, Fig. S2 *H*–*K*).

Together with biochemical evidence, we can tentatively correlate the global conformational space with the intersubunit communication when two GTP molecules bind simultaneously (Fig. 2*J*): When the second GTP molecule occupies the nucleotide binding pocket, it destabilizes the closed conformation and induces active communication between the subunits. These effects result in an open conformation, where the later-bound GTP is hydrolyzed. After hydrolysis, the Rag heterodimer returns to the single-GTP loaded configuration, and the global conformation returns to the closed state.

### Mutations Within the Nucleotide Binding Pockets Affect the Global Conformation of the Rag GTPases.

For canonical signaling GTPases, binding of different nucleotides shapes the conformation of the nucleotide binding pocket (local conformation) and directly determines its functional state. In the case of the Rag GTPases, however, we also need to consider the global conformation beyond the nucleotide binding pocket. To probe the potential allosteric effects, we investigated two frequently studied mutants, RagA-RagC(S75N) and RagA(T21N)-RagC. The mutated residues localize near the phosphate moiety of the bound nucleotide (*SI Appendix*, Fig. S4 *A* and *B*), and previous biochemical studies have shown that the asparagine substitution leads to a specific loss of GTP binding, but not GDP binding, to the corresponding subunit (5, 27). As a consequence, these two mutants cause strong mTORC1 signaling phenotypes in cells: RagA-RagC(S75N) hyperactivates mTORC1, while RagA(T21N)-RagC constantly inhibits mTORC1 (5, 6).

**Fig. 3.**
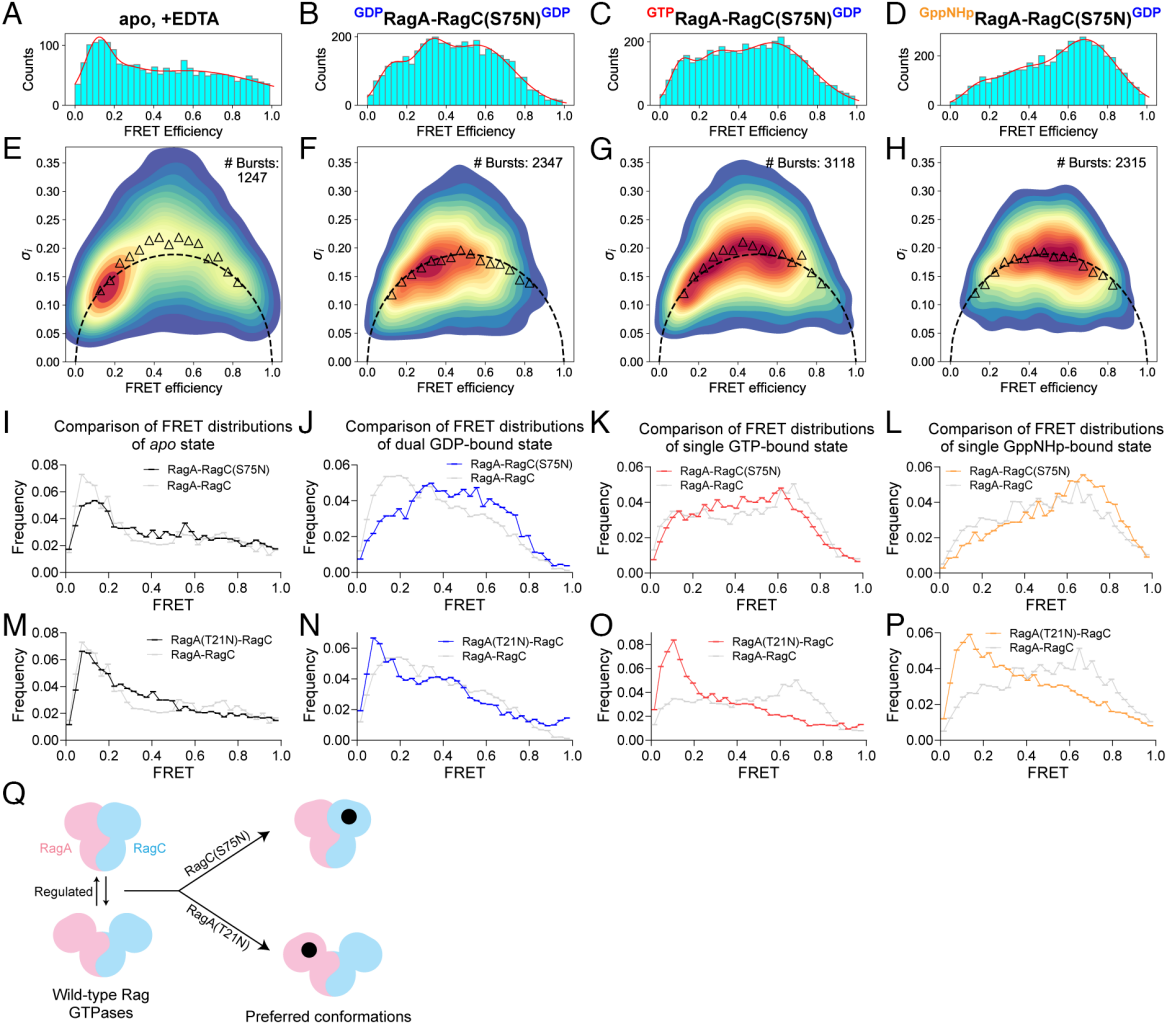
Mutations in the nucleotide binding pocket of the Rag subunit couple local changes with the global conformation of the heterodimer. (*A*–*D*) FRET distribution of RagA-RagC(S75N) in apo (*A*), dual GDP-loaded (*B*), GTP-GDP (*C*), and GppNHp-GDP (*D*) nucleotide loading configuration. (*E*–*H*) BVA of RagA-RagC(S75N) in apo (*E*), dual GDP-loaded (*F*), GTP-GDP (*G*), and GppNHp-GDP (*H*) nucleotide loading configuration. (*I*–*L*) Comparison of FRET distributions of wild-type RagA-RagC and RagA-RagC(S75N) in apo (*I*), dual GDP-loaded (*J*), GTP-GDP (*K*), and GppNHp-GDP (*L*) nucleotide loading configuration. (*M*–*P*) Comparison of FRET distributions of wild-type RagA-RagC and RagA(T21N)-RagC in apo (*M*), dual GDP-loaded (*N*), GTP-GDP (*O*), and GppNHp-GDP (*P*) nucleotide loading configuration. (*Q*) Summary of global conformational changes induced by local mutations within the nucleotide binding pocket.

We determined the global conformations of these two mutants under different nucleotide loading conditions. For the RagA-RagC(S75N) mutant, we observed a similar FRET distribution and dynamics to wild-type Rag GTPases in the apo state, as they both show a wide-open conformation, reflected by the dominant low-FRET population on the histogram (Fig. 3 *A*, *E*, and *I*). Strikingly, in the dual-GDP loaded configuration, RagA-RagC(S75N) displays much higher medium- and high-FRET populations in comparison to wild-type Rag GTPases under the identical condition (Fig. 3 *B*, *F*, and *J*). This observation suggests that the S75N mutation on RagC not only changes the nucleotide binding preference on the local scale but also induces a closure of the global conformation even in the wrong nucleotide loading configuration. In comparison, when the RagA subunit within the RagA-RagC(S75N) heterodimer binds GTP or GppNHp, a high-FRET population dominates (Fig. 3 *C*, *D*, *G*, *H*, *K*, and *L*), which is similar to the case of wild-type Rag GTPases. As RagA-RagC(S75N) causes mTORC1 hyperactivation in cells by enhancing the binding to the Raptor subunit (5, 32, 33, 45), our single-molecule results here suggest the S75N mutation may preposition the Rag GTPases into an optimized, closed conformation to interact with mTORC1.

When we performed similar experiments with RagA(T21N)-RagC, we were surprised by a complete decoupling of the global conformation from nucleotide loading. Under all the nucleotide loading configurations we tested, a predominant low-FRET population was observed across the board (Fig. 3 *M*–*P* and *SI Appendix*, Fig. S4 *C*–*J*), suggesting the RagA(T21N)-RagC mutant mainly resides in a wide-open conformation regardless of the bound nucleotides. Together with the data above, our results here suggest that point mutations within the nucleotide binding pocket, in addition to their local effect, may also govern the global conformation of the Rag GTPases (Fig. 3*Q*).

### mTORC1 Recruitment Induces Conformational Changes in the Rag GTPase Heterodimer.

**Fig. 4.**
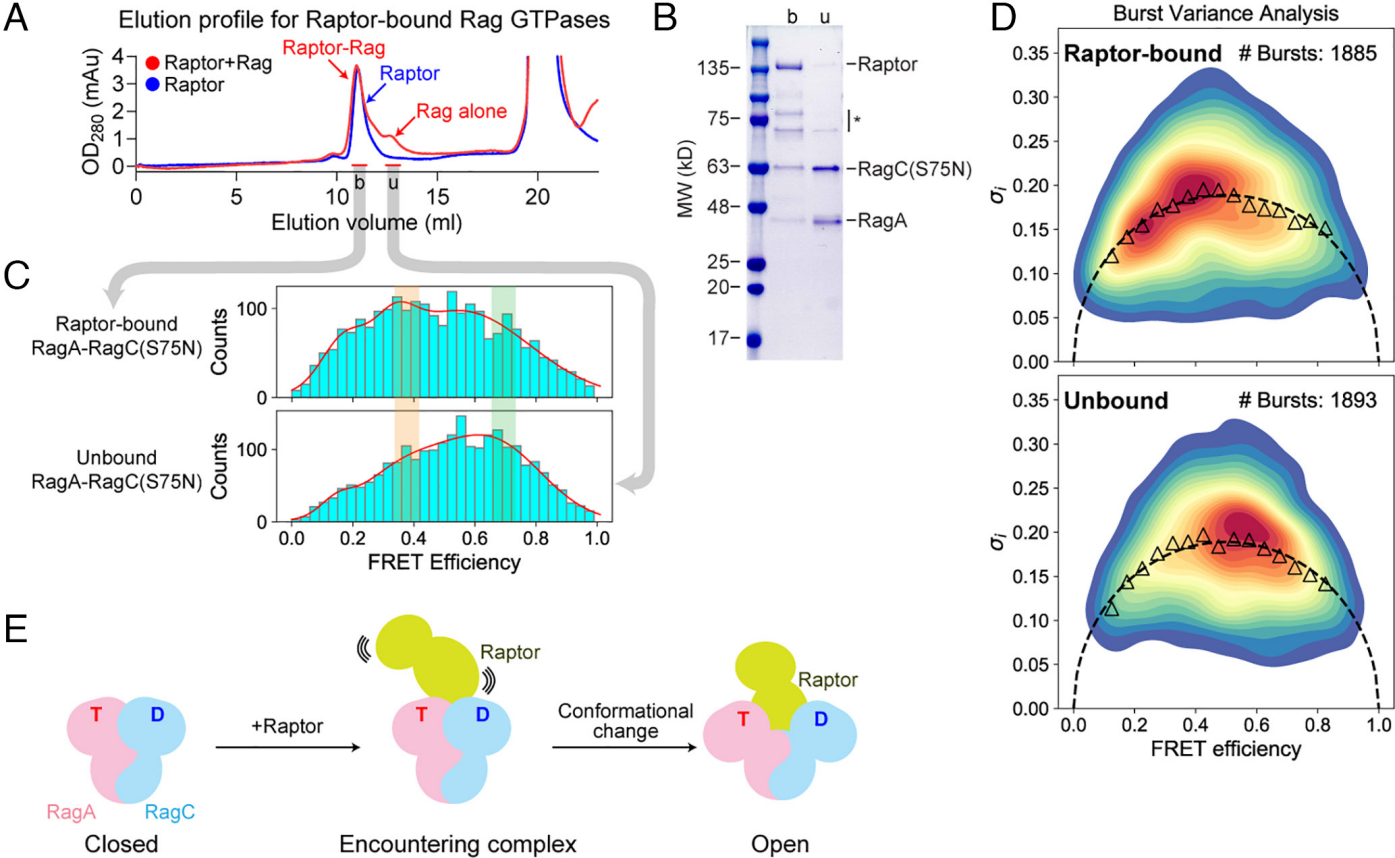
Binding to the downstream effector contracts the global conformational space. (*A*) Elution profile for Raptor alone (blue) and Raptor bound with Rag GTPases (red) on a gel-filtration column. (*B*) Coomassie-stained gel for the two fractions (b and u) in panel *A*. The Rag GTPases are observed in both fractions. The large molecular weight fraction (b) represents Raptor-bound Rag GTPases, while the small molecular weight fraction (u) represents free (unbound) Rag GTPases. The asterisk denotes commonly observed contaminants during Raptor purification. (*C*) FRET distributions for Raptor-bound (*Upper* panel) and unbound Rag GTPases (*Lower* panel). (*D*) BVA for Raptor-bound and unbound Rag GTPases. (*E*) An encountering complex between the Rag GTPases and Raptor serves as an on-pathway intermediate before stable interactions are established.

When amino acids are abundant, the Rag GTPase heterodimer binds to the Raptor subunit of mTORC1 and recruits the complex to the lysosomal surface. In the cryo-EM structural model of the Raptor–Rag–Ragulator cocomplex, the Rag GTPase heterodimer shows an open conformation (32, 33) (cf. *SI Appendix*, Fig. S1*A*). However, in our smFRET measurement, the activated state of the Rag GTPase heterodimer occupies a closed conformation (cf. Fig. 3*D*), suggesting a shift of the global conformation during mTORC1 recruitment. To get insight into this process, we included Raptor in our smFRET assay. We first incubated the activated form of Rag GTPases, ^GppNHp^RagA-RagC(S75N)^GDP^, with Raptor. To ensure we specifically measure the conformation of the Rag GTPases that bind to Raptor, we applied the mixture to a gel-filtration column (Fig. 4*A*). The Rag GTPases were detected at two positions: An earlier-eluted fraction corresponding to Raptor-bound Rag GTPases (Fig. 4*A*, “b”, bound), and a later-eluted fraction corresponding to the Rag GTPases alone (Fig. 4*A*, “u”, unbound). We confirmed the identity of each fraction on a Coomassie-stained gel (Fig. 4*B*) and proceeded with smFRET measurements. For the unbound Rag GTPases, we obtained a similar FRET profile as before (Fig. 4 *C*, *Bottom* panel) with a predominantly high FRET population. For the Raptor-bound fraction, however, the medium FRET population was heavily populated while the high FRET population diminished (Fig. 4 *C*, *Top* panel). Here, the medium FRET population has a FRET value of ~0.40, corresponding to a distance of 58 Å, which resembles the cryo-EM structural model (50 Å between the α-carbons of RagA-Glu98 and RagC-Met151). Moreover, this medium FRET population is a stable conformation, as the SD of *E*_sub_s from the experiment closely follows the static limit in BVA (Fig. 4*D*, triangle vs. dashed semicircle). These results suggest that mTORC1 reshapes the global conformation of the Rag GTPase heterodimer upon binding, and the closed conformation of the Rag GTPases is likely optimal for recruiting mTORC1 but not so for stable binding (Fig. 4*E*, see *Discussion*).

### A Critical Proline Residue Mediates Global Conformational Change.

**Fig. 5.**
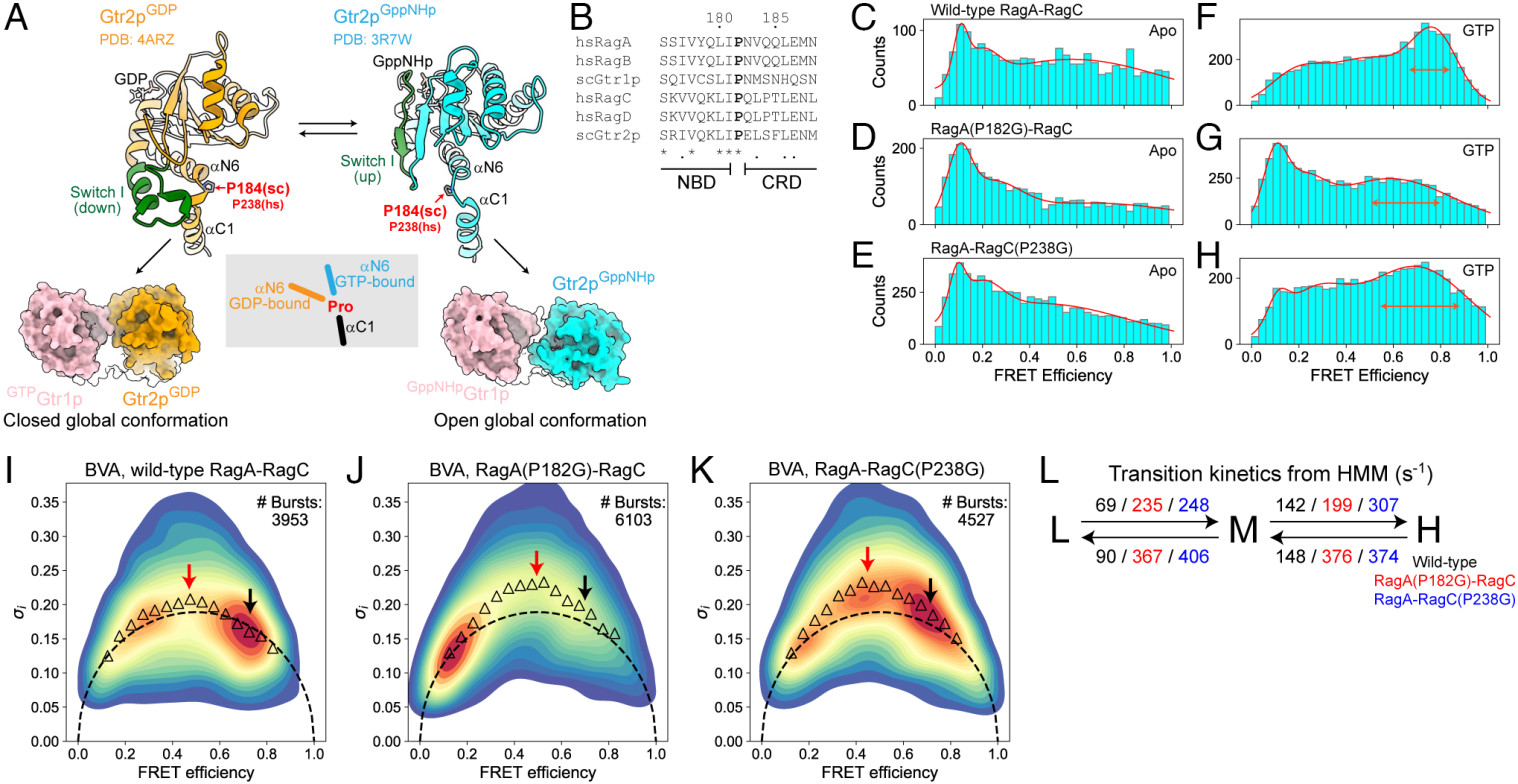
A critical proline residue mediates global conformation of the Rag GTPases. (*A*) Comparison of the conformation of Gtr2p (RagC homolog in *S. cerevisiae*) in GDP-bound (*Left* panel) vs. GppNHp-bound (*Right* panel) state. The first helix of CRD (αC1) is aligned for comparison while the rest of CRD is hidden for clarity. Pro184 is marked in red and shows different conformations in the two structural models. (*B*) Sequence alignment of the proline residue that mediates global conformational change. (*C*–*E*) FRET distribution of wild-type Rag (*C*), RagA(P182G)-RagC (*D*), and RagA-RagC(P238G) (*E*) in the apo state. (*F*–*H*) FRET distribution of wild-type Rag (*F*), RagA(P182G)-RagC (*G*), and RagA-RagC(P238G) (*H*) in single GTP-bound nucleotide loading configuration. (*I*–*K*) BVA of wild-type Rag (*I*), RagA(P182G)-RagC (*J*), and RagA-RagC(P238G) (*K*) in single GTP-bound nucleotide loading configuration. (*L*) Transition rates between the FRET states for wild-type Rag (black), RagA(P182G)-RagC (red), and RagA-RagC(P238G) (blue).

We next investigated the molecular basis that dictates the global conformation of the Rag GTPases and its biological relevance. As the CRDs of RagA and RagC tightly heterodimerize while their NBDs fluctuate, we paid close attention to the “hinge” region connecting the NBD to the CRD. Immediately, we noticed that a proline residue that bridges the last α-helix of NBD (αN6) to the first α-helix of CRD (αC1) shows distinct conformations in two crystal structural models: When Gtr2p (RagC homolog in *S. cerevisiae*) binds GDP, its Switch I adopts a downward (relaxed) conformation and pushes the Pro184 residue outward (29) (Fig. 5 *A*, *Left* panel). In contrast, when Gtr2p binds GppNHp (a GTP analogue), its Switch I adopts an upward conformation, allowing the Pro184 residue to flip inward (28) (Fig. 5 *A*, *Right* panel). As a consequence, the relative positioning of NBD in the structural model is directed toward different orientations based on the bound nucleotide (Fig. 5 *A*, *Middle* cartoon), allowing for a specific global conformation. Furthermore, this proline residue is evolutionarily conserved in both RagA and RagC in all eukaryotic homologs (Fig. 5*B*), so we decided to carry out structure–function analysis.

We mutated Pro182 of RagA or Pro238 of RagC to a glycine residue (termed as the “hinge mutation” below). In theory, such substitution should generate a more flexible hinge based on the Ramachandran plot and could potentially affect the global conformation. As controls, we first tested whether these mutations have any effect on the local conformation of individual nucleotide binding pockets by measuring the binding affinity of GTP and GDP using the radioactive crosslinking assay (*SI Appendix*, Fig. S5 *A*–*J*). Indeed, neither mutation has any major impact on the binding affinity (<twofold change compared with wild-type, *SI Appendix*, Fig. S5*K*). We next tested their ability to hydrolyze GTP. Under single turnover conditions, GTP hydrolysis by both mutants can be readily stimulated by GATOR1, a GAP for RagA (*SI Appendix*, Fig. S5 *L* and *M*), and by FLCN–FNIP2, a GAP for RagC (*SI Appendix*, Fig. S5 *N* and *O*), suggesting the nucleotide binding pocket of the hinge mutants can be correctly recognized by upstream regulators. These results suggest the glycine substitution maintains the local function of the nucleotide binding pocket, and their potential effect, if any, may be related to the modulation of the global conformation of the Rag GTPases (see below).

To directly probe the change of the global conformation, we carried out smFRET measurement with the hinge mutants, and observed striking differences (Fig. 5 *C*–*H*). In the single-GTP loaded state, the high FRET population is significantly lower and broader with RagA(P182G)-RagC (Fig. 5*G*) in comparison to wild-type Rag GTPases (Fig. 5*F*). Under the same condition, RagA-RagC(P238G) also shows a shifted FRET distribution, with a prominent medium-FRET population (Fig. 5*H*). This effect is specific to GTP (i.e., the closed conformation), as in the apo state (Fig. 5 *C*–*E*) and in the GDP-bound state (*SI Appendix*, Fig. S6 *A–C*), the hinge mutants behave similarly to wild-type Rag GTPases. Taken together, these results suggest a more flexible hinge distances the two NBDs and causes a more open global conformation.

In the FRET histograms, we noticed a significant broadening of the medium- and high-FRET populations (Fig. 5 *F*–*H*, horizontal arrows), implicating a potential increase in the dynamics. To test this hypothesis, we analyzed the data using BVA and HMM. For the medium-FRET population, the SD of *E*_sub_s is significantly higher for the hinge mutants (Fig. 5 *J* and *K*, red arrows) than for the wild-type Rag GTPases (Fig. 5*I*, red arrow). For the high-FRET population, while wild-type Rag GTPases show a static conformation (Fig. 5*I*, black arrow), the hinge mutants show more dynamic in-burst variations (Fig. 5 *J* and *K*, black arrows). HMM further validated this dynamic behavior, as the transition rates in between the FRET states are much faster with the hinge mutants (Fig. 5*L*). Therefore, in addition to a more open global conformation, hinge mutations also induce dynamics in the Rag GTPases as they likely fail to securely lock the closed global conformation when a single GTP binds (*SI Appendix*, Fig. S6 *D* and *E*).

**Fig. 6.**
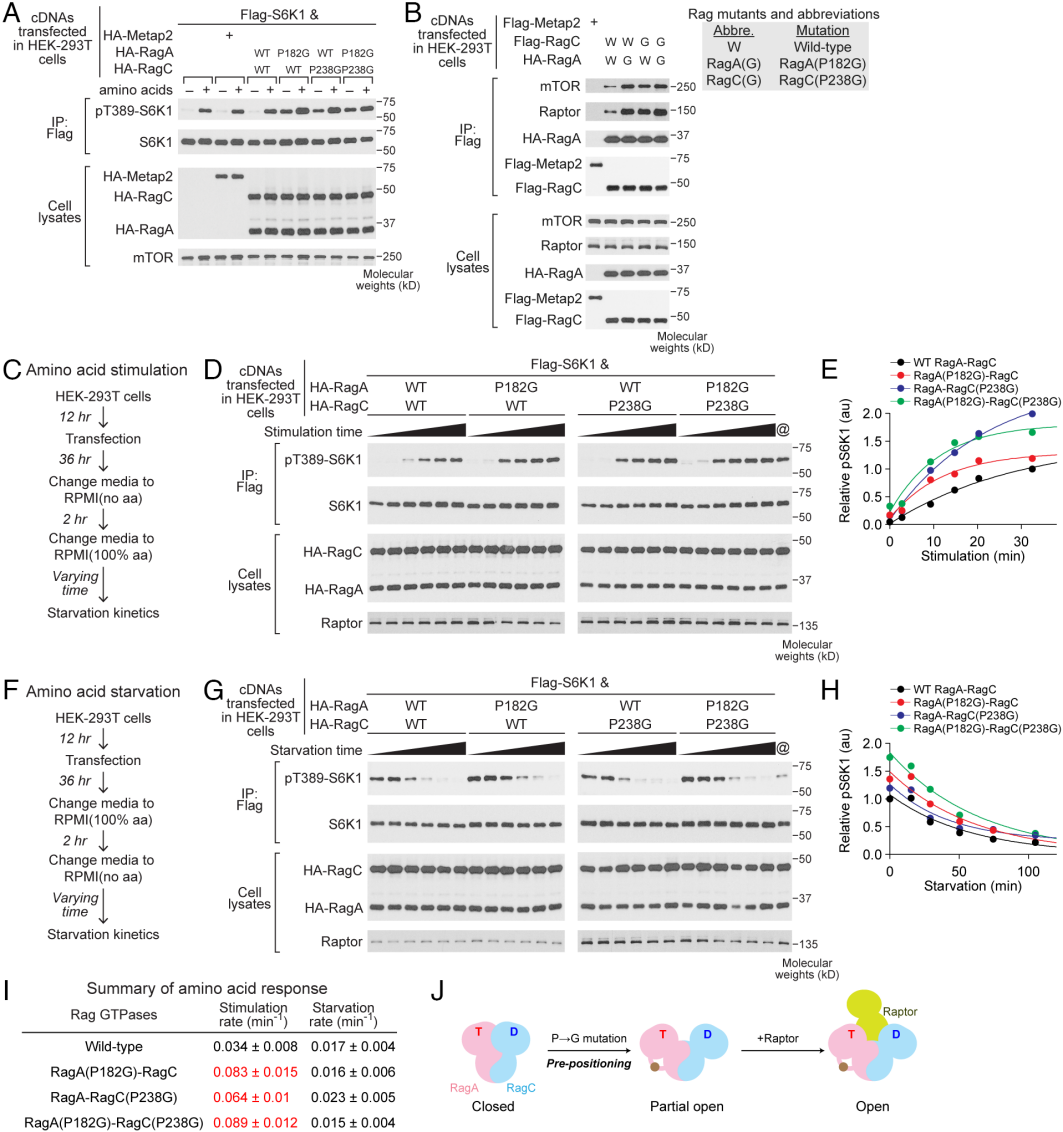
Modulating the global conformation of the Rag GTPase heterodimer alters mTORC1 signaling in cells. (*A*) Amino acid-regulated mTORC1 signaling mediated by the Rag GTPases. Rag GTPases carrying the hinge mutations enhance mTORC1 signaling in HEK-293 T cells. (*B*) Coimmunoprecipitation using Flag-tagged RagC to pull down HA-tagged RagA and endogenous mTORC1 subunits. Rag GTPases carrying the hinge mutations interact more strongly with mTORC1 in HEK-293 T cells. (*C*) Experiment to measure amino acid stimulation mediated by the Rag GTPases. (*D*) Time-course of Rag-mediated amino acid stimulation. (*E*) Quantification of the pT389-S6K1 signal in panel *D*. Stimulation rates are summarized below. (*F*) Experiment to measure amino acid starvation mediated by the Rag GTPases. (*G*) Time-course of Rag-mediated amino acid starvation. (*H*) Quantification of the pT389-S6K1 signal in panel *G*. Starvation rates are summarized below. (*I*) Summary of amino acid response. Reaction rates were reported as mean±SEM from three independent experiments. (*J*) Model for the effect of the hinge mutants. By partially opening up the global conformation, Rag GTPases carrying the hinge mutation bypass the encountering complex and facilitate Raptor binding, leading to a faster amino acid stimulation rate.

Finally, we sought to investigate the physiological consequence of modulating the global conformation of the Rag GTPases. We transiently expressed the hinge mutants in HEK-293 T cells and treated the cells with media deprived of or supplemented with amino acids. For wild-type Rag GTPases, the cells are sensitive to amino acids, as reflected by the regulated levels of phosphorylation of S6K1 on the Thr389 residue (pT389-S6K1, Fig. 6*A*, lanes 1–6). In comparison, the hinge mutants show elevated levels of pT389-S6K1 compared with wild-type Rag GTPases under the same condition (Fig. 6*A*, lanes 7–12 vs. lanes 5 and 6), suggesting these mutants distort nutrient signals from upstream sensors to mTORC1. In addition, when we performed a coimmunoprecipitation assay using the hinge mutants as baits, we pulled down a higher amount of mTORC1 (Fig. 6*B*), which is consistent with the signaling effect. As the hinge mutants preserve the local function of the nucleotide binding pocket while specifically disrupting the global conformation of the Rag GTPases (cf. Fig. 5 and *SI Appendix*, Fig. S5), these results imply a critical role of the global conformation during amino acid sensing in cells.

To further investigate the molecular mechanism, we performed a kinetic analysis of amino acid sensing mediated by the hinge mutants. Here, we tracked the activation (Fig. 6 *C*–*E*) or inactivation (Fig. 6 *F*–*H*) process of mTORC1 signaling in response to amino acid stimulation or starvation. We quantified the levels of pT389-S6K1 at different time points (Fig. 6 *D* and *G*) and calculated the activation and inactivation rates (Fig. 6 *E* and *H*). When the amino acid signal is mediated by the hinge mutants, we observed a significant increase in the activation rates (Fig. 6 *E* and *I*), while the inactivation rates remain largely the same (Fig. 6 *H* and *I*) compared with wild-type Rag GTPases. Combining with the in vitro data, these results suggest that the hinge mutants likely preposition the Rag GTPases into a partial open conformation that is suitable for Raptor binding (Fig. 6*J*), thus hyperactivate the mTORC1 pathway via bypassing the encountering checkpoint (cf. Fig. 4).

## Discussion

GTPases are considered as molecular switches in cells. By binding to different nucleotides, they show different conformations, allowing for regulated binding to the downstream effectors, thus gating the signaling pathway. Canonical signaling GTPases usually function in their monomeric form. Upon nucleotide binding, the Switch I and II regions around the nucleotide binding pocket adjust their conformations locally. In the amino acid sensing system, however, the heterodimerized Rag GTPases must coordinate the nucleotide loading on both subunits in a manner that extends beyond the local nucleotide binding pockets. Here, we used smFRET to visualize the global conformation of the Rag GTPases, as well as how nucleotide loading couples local conformations to the global conformation. Furthermore, we have identified a conserved proline residue that mediates the global conformational change. Our results reveal a regulatory mechanism in the amino acid sensing process in eukaryotic cells.

**Fig. 7.**
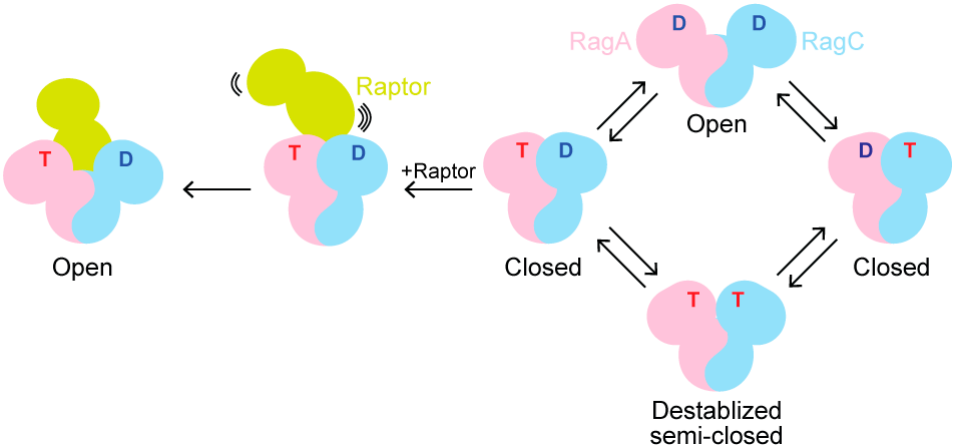
Summary of the global conformational space of the Rag GTPase heterodimer.

Mutations in the Rag GTPases are frequently studied because they are often correlated with diseases and are relevant for biochemical assays (Table 1). They can trap the Rag GTPases in defined functional states to achieve controlled manipulation of the downstream mTORC1 signaling. Based on our results here, we can tentatively classify the mutants into two categories. First, as represented by RagA(T21N) and RagC(S75N), these mutants shift the nucleotide binding preference by abolishing GTP binding, but not GDP binding, to the corresponding subunit. Such local effects lead to a modulation of the global conformation, with RagA(T21N) promoting the open conformation while RagC(S75N) promoting the closed conformation. The second class of mutants is global conformation-specific, represented by RagA(P182G) and RagC(P238G). These mutants maintain the local function of the nucleotide binding pocket, but specifically disrupt the global conformation. In both cases, mTORC1 signaling is distorted, suggesting both local and global conformations are critical for correctly interpreting amino acid signals (Fig. 7).

**Table 1.** Summary of Rag GTPase mutants and their behavior

Mutants	Nucleotide binding preference	Global conformation (GTP-loaded)	Effect on mTORC1 signaling
RagA(T21N)	Impairs GTP binding but not GDP binding to RagA	Predominant low FRET	Inhibits mTORC1 activity
RagC(S75N)	Impairs GTP binding but not GDP binding to RagC	Increased prevalence of the high FRET population	Stimulates mTORC1 activity
RagA(P182G)	No effect	Predominant low FRET	Faster mTORC1 activation
RagC(P238G)	No effect	Decreased high FRET population	Faster mTORC1 activation

During mTORC1 activation, the Rag GTPases are singly loaded with GTP on RagA, which generates a closed global conformation. However, in the structural model when mTORC1 is stably bound, the Rag GTPases show an open conformation, in which the “claw” of Raptor inserts in between the Rag subunits to make additional contacts to secure binding. This discrepancy suggests a multistep binding event when the Rag GTPases recruit mTORC1. Here, we propose an “encountering complex”, defined when Raptor initially touches the Rag GTPases, exists and it has a different conformation than in the stably bound Raptor–Rag complex (Fig. 7). Similar encountering complexes have been observed in other systems. For example, during cotranslational protein targeting, the signal recognition particle (SRP) and its receptor first form an “early complex” in a nucleotide-independent manner, before rearranging into a stable “closed complex” to deliver the ribosome to the translocon (46, 47). This transient encountering complex usually encodes additional regulatory checkpoint during protein–protein interaction. Identifying such interactions and observing the conformational rearrangements within the encountering complex will offer mechanistic insights into the activation of the mTORC1 pathway in the future.

## Materials and Methods

The Rag GTPase heterodimer and the double-cysteine mutants were purified using an established protocol (48). Fluorescently labeled Rag GTPase heterodimer was prepared using Cy3 and Cy5-maleimide (Cytiva). Alternating laser excitation (ALEX)-based single-molecule FRET was carried out on an EI-FLEX single-molecule fluorescence spectrometer (Exciting Instruments). Single-molecule data were processed using the FRETBursts software package (49) (see *SI Appendix* for details).

## Supplementary Material

Appendix 01 (PDF)

## Data Availability

All study data are included in the article and/or *SI Appendix*.

## Footnotes

^*^Ideally, we would purify individual Rag subunits, label them individually with Cy3 or Cy5, and then assemble the heterodimer. However, this is technically impossible as the CRD contains many hydrophobic residues at the dimerization interface, which strongly induce aggregation when expressed alone. Currently, we can only purify the Rag heterodimer by coexpressing the two subunits and then perform statistical labeling.

^†^The distance (*R*) was estimated based on the reported Förster radius between Cy3 and Cy5 fluorophores (*R*_0_ = 56 Å) using the equation *E* = 1/[1 + (*R*/*R*_0_)^6^].

^‡^In this study, we follow the nomenclature defined by Harris et al. (42). In a frequency-based analysis (FRET histogram), we use “FRET populations.” In hidden Markov Modeling, we use “FRET states.” In the case of a static conformation, a FRET population equals a FRET state. In the case of multiple dynamic conformations, a FRET population is an average of two or more FRET states.
