# Supplementary material for: Global conformation of the Rag GTPase heterodimer governs eukaryotic amino acid sensing
Source: Proc Natl Acad Sci U S A. 2025 Oct 15;122(42):e2517050122. doi: 10.1073/pnas.2517050122 (PMC12557722; doi:10.1073/pnas.2517050122)
Supplement: Supplementary file 1 — Appendix 01 (PDF) [file pnas.2517050122.sapp.pdf]

PNAS
www.pnas.org

Supplementary Materials

Global conformation of the Rag GTPase heterodimer governs eukaryotic amino acid sensing

Dylan D. Doxsey[1] and Kuang Shen[1,2,*]

1 Program in Molecular Medicine, University of Massachusetts Chan Medical School, 366 Plantation St, Worcester, MA 01605, USA
2 Department of Biochemistry and Molecular Biotechnology, University of Massachusetts Chan Medical School, 364 Plantation St, Worcester, MA 01605, USA

* Corresponding author. E-mail: Kuang.Shen@umassmed.edu (K.S.)

Tel: 774-455-6604 (K.S.)

This document contains Materials and Methods, and Supplementary Figures 1-6 and legends.

Materials and Methods

Cell lines

HEK-293T cells were obtained from ATCC and were maintained in a ThermoFisher Forma CO_2 incubator set at 37 °C, 5% CO_2, and 100% humidity. They were cultured in DMEM supplemented with 10% inactivated bovine serum (Corning),100 IU/ml penicillin, and 100 μg/ml streptomycin.

BL21(DE3) *E. Coli* strains were grown at 37 °C in a New Brunswick S44i shaker in LB media. To induce protein expression, they were transformed by the corresponding plasmids, propagated at 37 °C, and induced with IPTG at 18 °C.

Protein purification

The Rag GTPase heterodimer was purified as previously described[49]. In brief, co-expression of His-tagged RagA and tagless RagC was induced by 0.5 mM IPTG in BL21(DE3) cells when the optical density (OD) of the culture reaches 0.6. The Rag GTPase heterodimer was purified sequentially through Ni-NTA (Qiagen), MonoQ (Cytiva), and Superose 6 (Cytiva) columns. For the Rag GTPase mutants, point mutation was introduced using QuikChange (Agilent), and the mutants were purified similarly to wild-type. Cyslite version of the Rag GTPase heterodimer contains the following point mutations on RagA: C124V, C157L, C159A, C219S, and on RagC: C52S, C286S, C297D, C339A, C358A, C377S.

Raptor was purified as previously described[32]. In brief, Flag-tagged human Raptor was transiently transfected and expressed in FreeStyle 293-F cells (Thermo). Sixty hours post transfection, cells were harvested and lysed with CHAPS Lysis Buffer [CLB, 40 mM NaHEPES, pH 7.4; 5 mM $MgCl_2$; 10 mM $Na_4P_2O_7$; 10 mM Na β-glycerophosphate; 0.3% CHAPS; and 1:100 protease inhibitor (GoldBio)]. Flag-Raptor was pulled down using Flag-M2 gel (Sigma), and eluted with Flag peptide, before further purification using gel-filtration chromatography.

Fluorescent labeling

Fluorescently labeled Rag GTPase heterodimer was prepared using Cy3 and Cy5-maleimide (Cytiva). Cysteine residues on the Rag GTPase heterodimer were first reduced by 0.5 mM TCEP for an hour at room temperature. The labeling reaction was carried out in a buffer containing 50 mM NaHEPES, pH 7.4, 100 mM NaCl, 2 mM $MgCl_2$, 0.5 mM TCEP, and 100 μM GDP for 2 hours at room temperature with gentle tumbling. The protein to dye ratio was 1:5:5 (Rag:Cy3:Cy5). Unconjugated reactive dyes were quenched by 20 mM DTT, and free dyes were separated from labeled proteins using gel-filtration chromatography.

Single molecule experiment

Alternating laser excitation (ALEX)-based single molecule FRET was carried out on a EI-FLEX single-molecule fluorescence spectrometer (Exciting Instruments) with the laser power set at 0.1 mW for 520 nm and 0.04 mW for 635 nm. The Rag GTPase heterodimer was incubated in Assay Buffer (50 mM NaHEPES, pH 7.4; 100 mM NaCl; 20 mM EDTA; 2mM DTT; 0.1% CHAPS) and corresponding nucleotides overnight. The following morning, 25 mM $MgCl_2$ was added to stabilize nucleotide binding, and the sample was ultracentrifuged at 200,000×*g* for 30 minutes to remove aggregations during processing. The Rag GTPase heterodimer was then diluted in Assay Buffer supplemented with corresponding nucleotides and $MgCl_2$. The dilution was dependent on the labeling efficiency and the resulting protein concentration after ultracentrifugation. Four to six 10-minute movies were collected for each sample.

Single molecule data processing

Frequency-based analysis was carried out using the FRETBursts package[48]. Movies were first combined and background was subtracted. Photon bursts were identified by FRETBursts[48]. Donor-only and acceptor-only bursts were then removed, and the FRET efficiency E and stoichiometry S were calculated using

the following equations: $E = \frac{f_{D_{EX}}^{A_{EM}}}{f_{D_{EX}}^{D_{EM}} + f_{D_{EX}}^{A_{EM}}}$, $S = \frac{f_{D_{EX}}^{D_{EM}} + f_{D_{EX}}^{A_{EM}}}{f_{D_{EX}}^{D_{EM}} + f_{D_{EX}}^{A_{EM}} + f_{A_{EX}}^{A_{EM}}}$, where $f_{D_{EX}}^{A_{EM}}$ is the number of acceptor photons during donor excitation after background subtraction; $f_{D_{EX}}^{D_{EM}}$ is the number of donor photons during donor excitation; $f_{A_{EX}}^{A_{EM}}$ is the number of acceptor photons during acceptor excitation[39]. Channel leakage was corrected for FRET efficiency. The γ-factor was assumed to be 1.0. Histograms of *E* and *S* were then plotted accordingly.

Burst variance analysis (BVA) was performed using the FRETBursts package[40,48], by calculating the standard deviation of the FRET efficiency of sub-bursts (E_{sub}s) within each burst, with a sliding window of seven photons. The static limit was calculated by the equation: $\sigma_E = \sqrt{\frac{E(1-E)}{n}}$, where *E* is the FRET efficiency and *n* equals seven here.

Hidden Markov modeling was performed using the burstH2MM[41,42] software package. This approach applies Hidden Markov Models (HMMs) to photon-by-photon fluorescence data in order to identify the hidden FRET states a protein transitions between. Photon arrival time streams were used as input, and only bursts containing more than 25 photons were selected for analysis to ensure reliability. For each dataset, models with increasing numbers of hidden states were constructed. Each candidate model was fit to the photon sequences, and the integrated complete likelihood (ICL) was used as the criterion to compare models. The optimal number of hidden states was defined as the point at which the ICL converged. Once the optimal model was identified, the Viterbi algorithm was applied to decode the most probable sequence of hidden states that generated the observed photon stream. Specifically, the Viterbi procedure computes, for every photon, the path through the hidden states that maximizes the joint probability of the observations and the state sequence. This is achieved through: (i) initialization with state priors, (ii) recursive propagation of maximum-likelihood paths across the photon sequence, and (iii) backtracking to recover the globally optimal state trajectory. As a result, each photon was assigned to its most likely hidden state, and photons were binned accordingly. From these state assignments, we quantified the photophysical properties of each state as well as the transitions between states. Transition rate matrices derived from the HMM allowed us to calculate the percent occupancy of each state, providing insight into the equilibrium distribution and kinetics of conformational dynamics.

Preparation of cell lysates and immunoprecipitation

sgRagA/B and sgRagC/D cell lines were generated in a previous study[24]. Cell lysates and immunoprecipitates were prepared as follows. Two million HEK-293T cells were plated onto a 10 cm petri dish. Twenty-four hours later, the cells were transfected with corresponding plasmids. Thirty-six hours later, HEK-293T cells were rinsed once with ice-cold PBS and lysed by Triton Lysis Buffer [TLB, 40 mM NaHEPES, pH 7.4; 5 mM $MgCl_2$; 10 mM $Na_4P_2O_7$; 10 mM Na β-glycerophosphate; 1% Triton X-100; and 1:100 protease inhibitor (GoldBio)]. The lysates were cleared by centrifuging at 20,000×*g* for 10 minutes and immunoprecipitated with Flag-M2 affinity gel. Following immunoprecipitation, Flag-M2 gel was washed once with TLB and three times with TLB supplemented with 500 mM NaCl. Immunoprecipitated proteins were denatured by 5×SDS loading buffer, resolved by SDS-PAGE, and analyzed by immunoblotting. Western blots were quantified by an LI-COR imaging system.

For amino acid stimulation and starvation experiment, cells were transfected with corresponding plasmids. The starvation condition was in RPMI media with no amino acids supplemented with 10% dialyzed serum, 100 IU/ml penicillin, and 100 µg/ml streptomycin. The stimulation condition was in regular RPMI media supplemented with 10% dialyzed serum, 100 IU/ml penicillin, and 100 µg/ml streptomycin. Cells were treated in these media for defined periods of time at 37 °C, before lysed and analyzed as above.

Kinetic and equilibrium binding assays

Nucleotide binding assay

Guanine nucleotide binding affinity to the Rag GTPase heterodimer was measured using an established crosslinking assay[27,49]. The Rag GTPase heterodimer was thawed on ice and ultracentrifuged at 200,000×*g* for 30 minutes to remove aggregation. The protein concentration was freshly taken using Bradford (BioRad). Equilibrium titrations were carried out in Assay Buffer (50 mM NaHEPES, pH 7.4; 100 mM potassium acetate; 2 mM $MgCl_2$; 2 mM DTT; 0.1% CHAPS). 5 nM α-^{32}P-GTP or α-^{32}P-GDP was incubated with Rag GTPase heterodimer ranging from 5 nM - 5 μM for 8 hours at 4 °C. The mixture was applied on a chilled aluminium block covered with parafilm, and was exposed to 0.3 Joule of ultraviolet light (Spectronics). The crosslinked reaction mixture was then mixed with 5×SDS loading buffer, boiled, and analyzed on a 10-20% tris-glycine gel. The gel was rinsed in SDS destaining solution, fixed, and dried. The dried gel was exposed to a phosphorimaging screen (Cytiva) overnight, which was imaged and the band intensities were quantified and fit to a single-site binding equation to extract the binding constant K_d.

Stimulated GTP hydrolysis assay

GTP hydrolysis reactions were carried out in Assay Buffer. 50 nM of the Rag GTPase heterodimer was preloaded with 0.1 nM of α-^{32}P-GTP. Increasing concentrations of GATOR1 or FLCN-FNIP2 were incubated with loaded Rag GTPase heterodimer. Small amounts of the reaction were aliquoted at various time points and quenched using 0.75 M KH_2PO_4 (pH 3.3). The time points were analyzed by thin layer chromatography (TLC) plates, and imaged and quantified using a phosphorimager (Cytiva). Linear regression was used to fit the fraction of hydrolyzed GTP against time, to generate the observed rate constants (k_{obsd}) corresponding to each concentration. The k_{obsd}s were fit to a single-site binding equation to extract the k_{cat} and K_m values.

Supplementary Figure and Legends

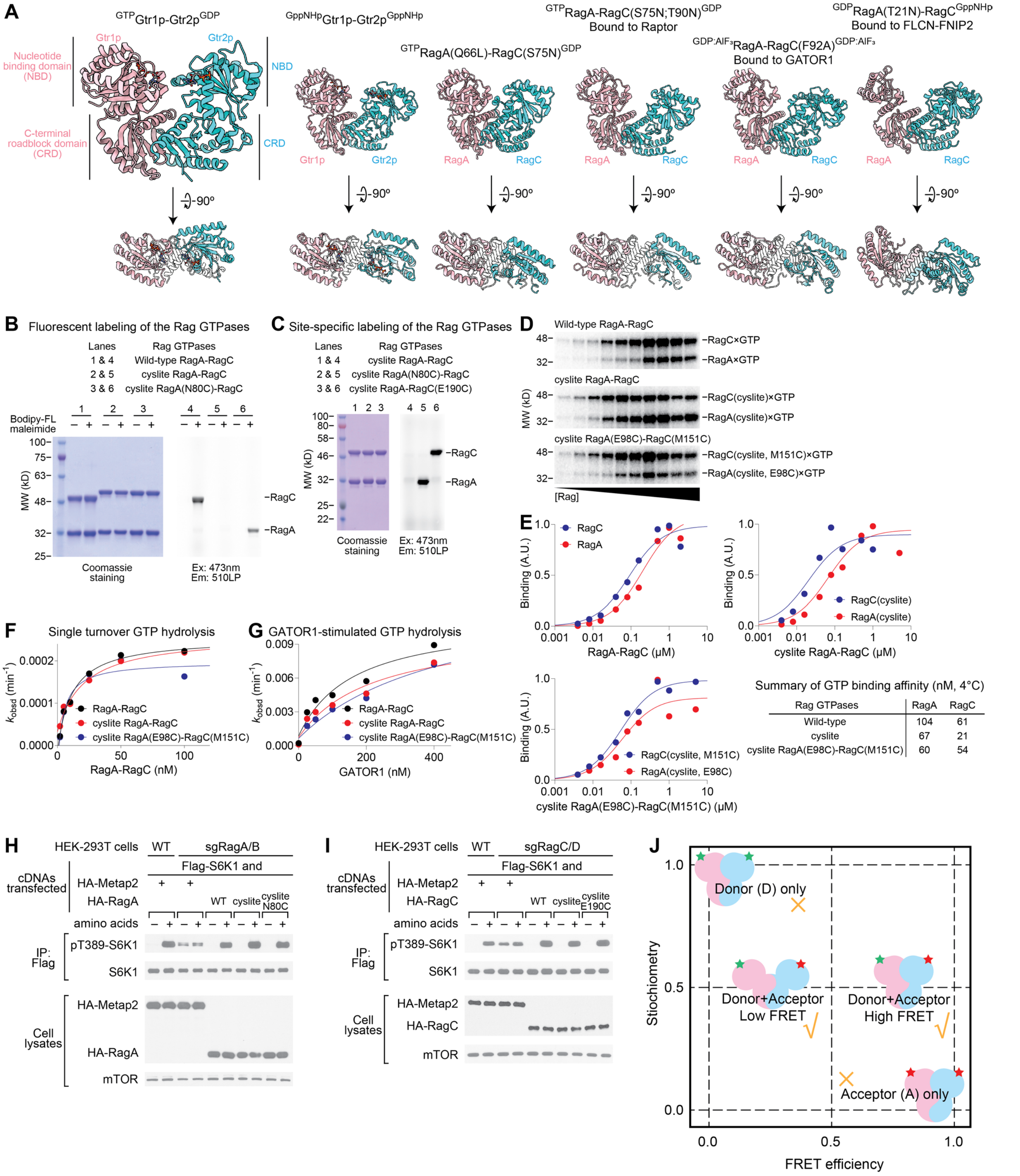

Figure S1. Fluorescent labeling of the Rag GTPase heterodimer.

A. The nucleotide binding domains of the Rag GTPase heterodimer show different conformations under different conditions. The models are aligned to the CRD of RagA/Gtr1p subunit within each heterodimer, and the nucleotide binding domain of RagC/Gtr2p shows dramatically different conformations depending on the nucleotide loading configuration, the mutation it carries, and the binding partner. From left to right, the PDB codes for the structural models are: 4ARZ, 3R7W, 6S6D, 6U62, 7T3B, and 6ULG.

B. A cysteine-light (cyslite) version of the Rag GTPase heterodimer allows for fluorescent labeling in a site-specific manner. Wild-type Rag GTPase heterodimer carries natural cysteine residues that can be undesirably labeled (Lanes 1 and 4). Cyslite Rag GTPase heterodimer eliminates these residues and provides a clean background (Lanes 2 and 5). A cysteine residue can then be introduced at desired positions to allow for site-specific labeling (Lanes 3 and 6).

C. Site-specific labeling of the Rag GTPases with a fluorescent dye. Both RagA (Lanes 2 and 5) and RagC (Lanes 3 and 6) subunits can be labeled in a site-specific manner, while the cyslite background is clean (Lanes 1 and 4).

D. Radioactive crosslinking assay to measure the binding affinity of GTP to wild-type Rag GTPases, cyslite Rag GTPases, and the double cysteine mutant used for labeling.

E. Quantification of the binding affinity of GTP to the Rag GTPases. Wild-type Rag GTPases, cyslite Rag GTPases, and the double cysteine mutant used for labeling have similar binding affinities to GTP.

F. Single turnover hydrolysis assay to measure the GTP hydrolysis by wild-type Rag GTPases, cyslite Rag GTPases, and the double cysteine mutant used for labeling. They show similar $K_{½}$ and k_{cat}.

G. Stimulated hydrolysis assay to measure the hydrolysis rate of the Rag GTPases. GATOR1 stimulates GTP hydrolysis by wild-type Rag GTPases, cyslite Rag GTPases, and the double cysteine mutant used for labeling to a similar degree.

H. Cyslite RagA restores amino acid signals in RagA/B double knockout cells, similar to wild-type RagA.

I. Cyslite RagC restores amino acid signals in RagC/D double knockout cells, similar to wild-type RagC.

J. Schematic *E*-*S* plot differentiating different fluorescently-labeled protein molecules.

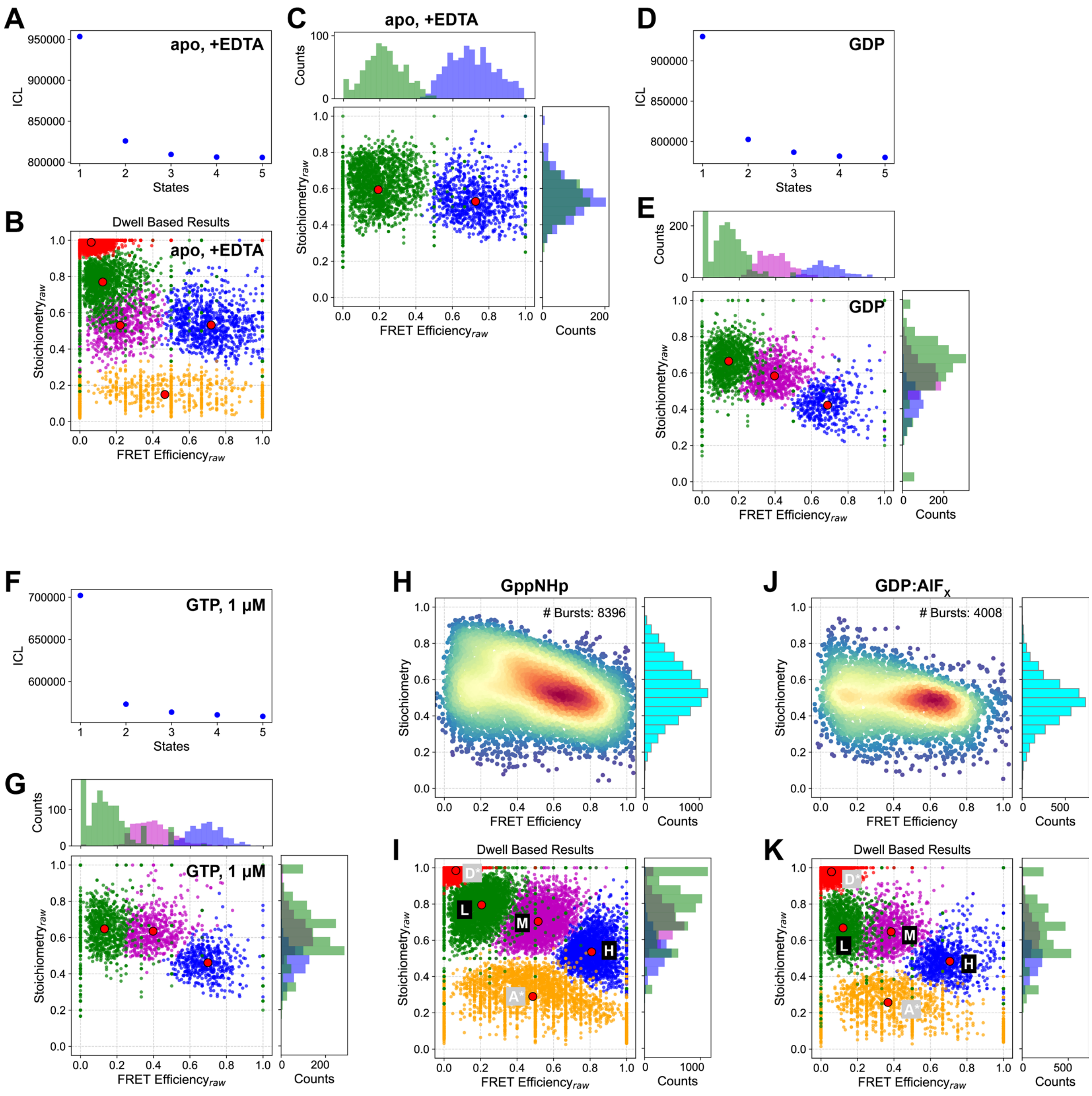

Figure S2. Global conformation of the Rag GTPase heterodimer detected by single molecule FRET.

A. Integrated completed likelihood (ICL) of HMM simulation of smFRET data. A four-state model is sufficient to describe the photon behavior of the apo state Rag GTPases.

B. A five-state HMM model generates two closely related FRET states (green and purple).

C. FRET and stoichiometry distribution of the **L** and **H** states, based on a four-state HMM model from smFRET data of apo Rag GTPases (Fig. 1E).

D. ICL of HMM simulation of smFRET data from dual GDP-loaded Rag GTPases.

E. FRET and stoichiometry distribution of the **L**, **M**, and **H** states, based on a five-state HMM model from smFRET data of dual GDP-loaded Rag GTPases (Fig. 1H).

F. ICL of HMM simulation of smFRET data from single GTP-loaded Rag GTPases.

G. FRET and stoichiometry distribution of the **L**, **M**, and **H** states, based on a five-state HMM model from smFRET data of single GTP-loaded Rag GTPases (Fig. 1K).

H. *E*-*S* diagram of the Rag GTPase heterodimer in dual GppNHp-loaded state.

I. A five-state HMM model of the Rag GTPase heterodimer in dual GppNHp-loaded state.

J. *E*-*S* diagram of the Rag GTPase heterodimer in dual GDP:AlF$_X$-loaded state.

K. A five-state HMM model of the Rag GTPase heterodimer in dual GDP:AlF$_X$-loaded state.

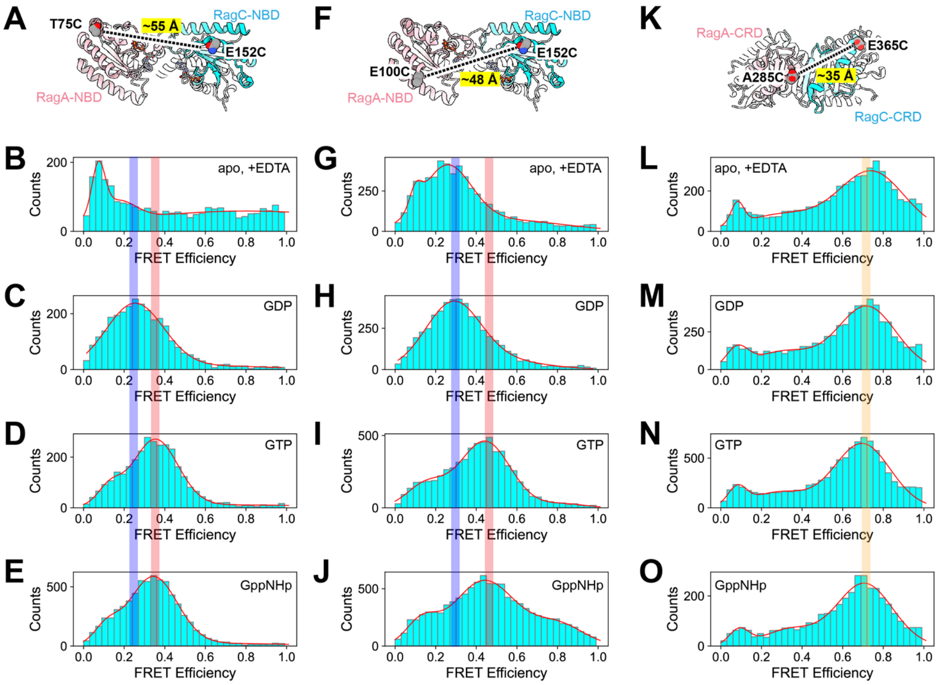

Figure S3. GTP-induced compaction of the global conformation of the Rag GTPase heterodimer.

A. Scheme of the FRET pair RagA(T75C)-RagC(E152C).

B-E. FRET distribution of Cy3/Cy5-labeled RagA(T75C)-RagC(E152C) in apo (B), GDP (C), GTP (D), or GppNHp (E) bound state.

F. Scheme of the FRET pair RagA(E100C)-RagC(E152C).

G-J. FRET distribution of Cy3/Cy5-labeled RagA(E100C)-RagC(E152C) in apo (G), GDP (H), GTP (I), or GppNHp (J) bound state.

K. Scheme of the FRET pair RagA(A285C)-RagC(E365C).

L-O. FRET distribution of Cy3/Cy5-labeled RagA(A285C)-RagC(E365C) in apo (L), GDP (M), GTP (N), or GppNHp (O) bound state.

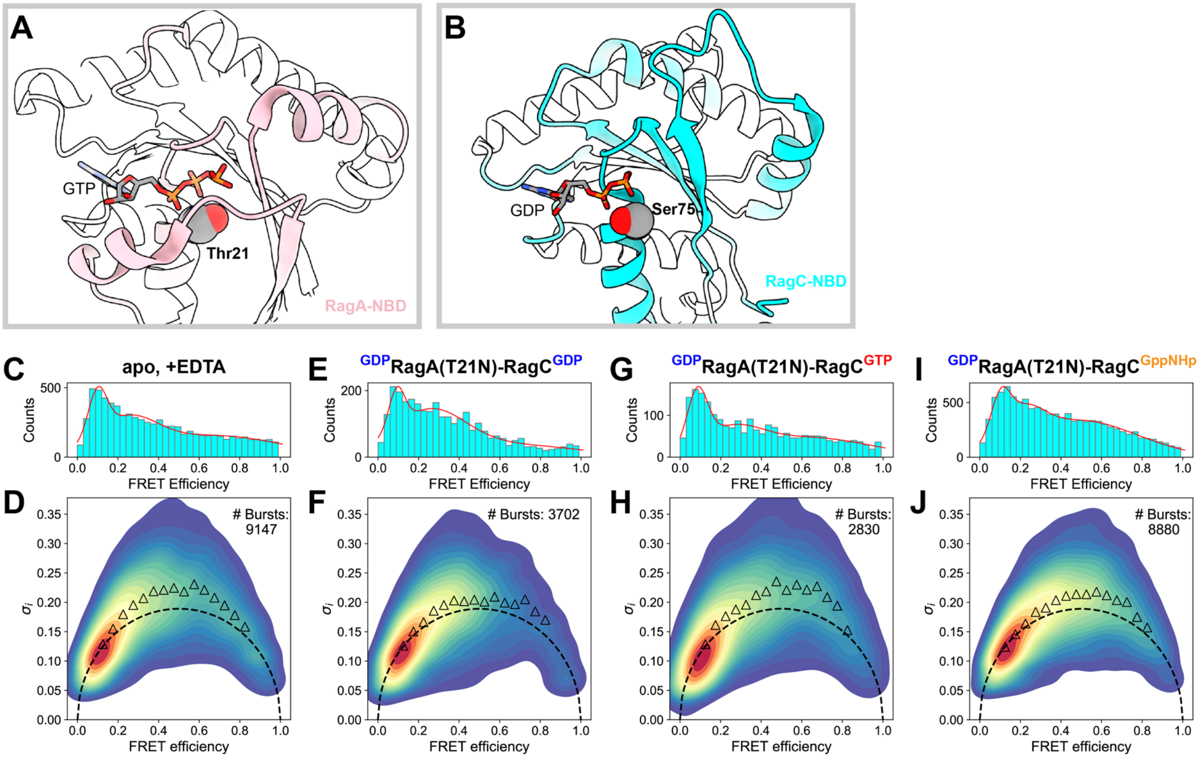

Figure S4. Point mutations within the nucleotide binding pocket couple local changes to the global conformation.

A & B. Position of Thr21 on RagA (A) and Ser75 on RagC (B) in crystal structural models.

C. FRET histogram of RagA(T21N)-RagC in apo state.

D. BVA of RagA(T21N)-RagC in apo state.

E. FRET histogram of RagA(T21N)-RagC in dual GDP-loaded state.

F. BVA of RagA(T21N)-RagC in dual GDP-loaded state.

G. FRET histogram of RagA(T21N)-RagC in single GTP-loaded state.

H. BVA of RagA(T21N)-RagC in single GTP-loaded state.

I. FRET histogram of RagA(T21N)-RagC in single GppNHp-loaded state.

J. BVA of RagA(T21N)-RagC in single GppNHp-loaded state.

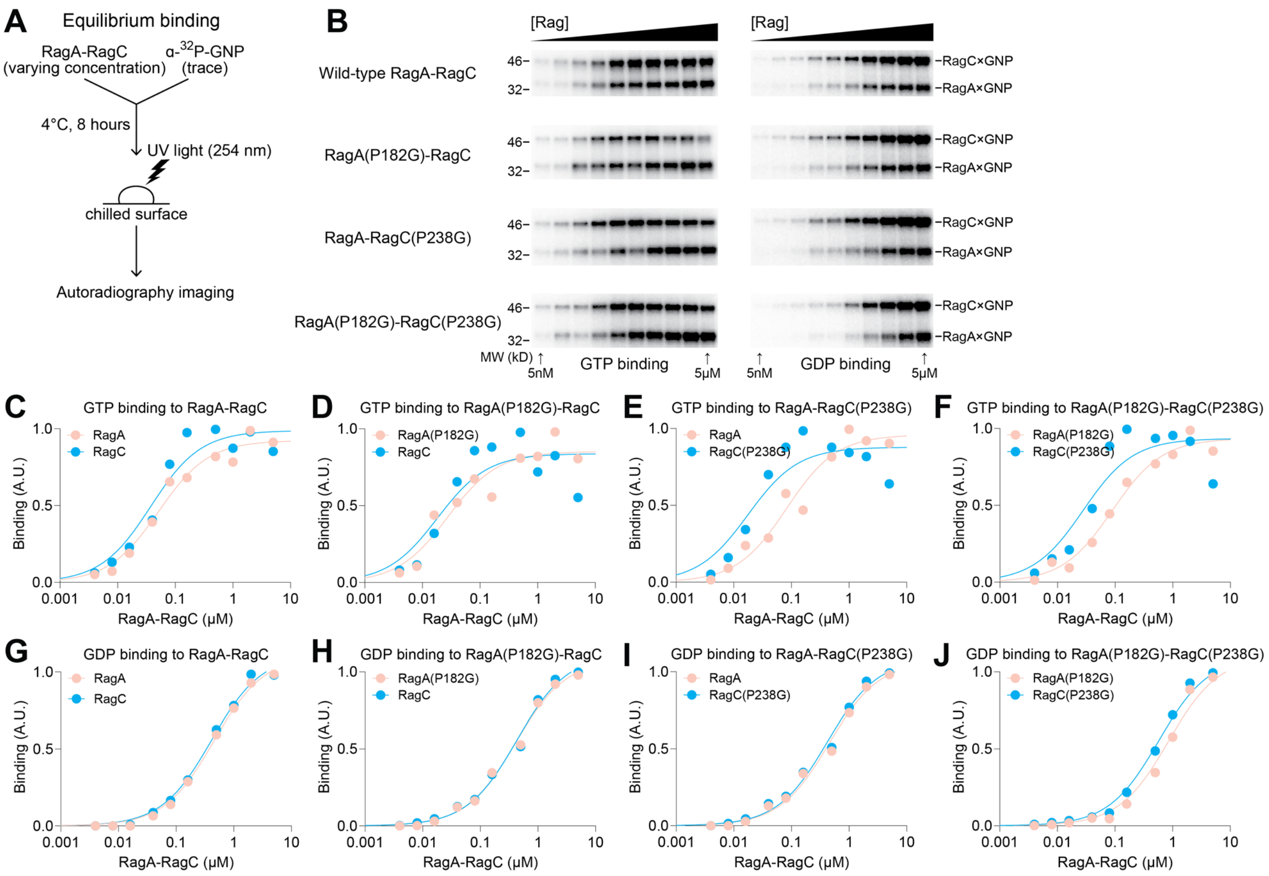

K Summary of nucleotide binding affinity (4°C)

K_d (nM)	RagA GTP	RagA GDP	RagC GTP	RagC GDP
Wild-type	51 (15)	455 (65)	37 (10)	410 (81)
RagA(P182G)-RagC	27 (9)	431 (49)	18 (8)	408 (55)
RagA-RagC(P238G)	83 (15)	450 (45)	18 (6)	416 (40)
RagA(P182G)-RagC(P238G)	89 (10)	860 (82)	28 (8)	560 (75)

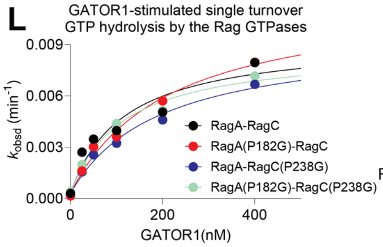

M Summary of kinetic parameters (25°C)

Substrate	k_{cat} (min^{-1})	$K_{½}$ (nM)
Wild-type	9.0×10^{-3} (1.5)	117 (20)
RagA(P182G)-RagC	1.2×10^{-2} (0.2)	215 (43)
RagA-RagC(P238G)	9.2×10^{-3} (1.6)	194 (45)
RagA(P182G)-RagC(P238G)	8.4×10^{-3} (1.3)	114 (35)

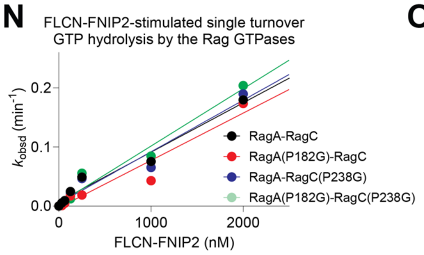

O Summary of kinetic parameters (4°C)

Substrate	$k_{cat}/K_{½}$ ($M^{-1}s^{-1}$)
Wild-type	1.4×10^{3} (0.1)
RagA(P182G)-RagC	1.3×10^{3} (0.2)
RagA-RagC(P238G)	1.5×10^{3} (0.1)
RagA(P182G)-RagC(P238G)	1.6×10^{3} (0.2)

Figure S5. Biochemical characterization of the hinge mutants.

A. Radioactive crosslinking assay to measure nucleotide binding affinity to Rag subunits.

B. Crosslinking of wild-type Rag GTPases, RagA(P182G)-RagC, RagA-RagC(P238G), and RagA(P182G)-RagC(P238G) to radioactively labeled GTP (left panels) or GDP (right panels).

C-J. Quantification of panel B to extract the binding constants.

K. Summary of binding constants. Numbers in grey are SEM from three independent assays.

L. GATOR1-stimulated GTP hydrolysis by wild-type Rag GTPases and mutants.

M. Summary of kinetic parameters in panel L. Numbers in grey are SEM from three independent assays.

N. FLCN-FNIP2-stimulated GTP hydrolysis by wild-type Rag GTPases and mutants.

O. Summary of kinetic parameters in panel N. Numbers in grey are SEM from three independent assays.

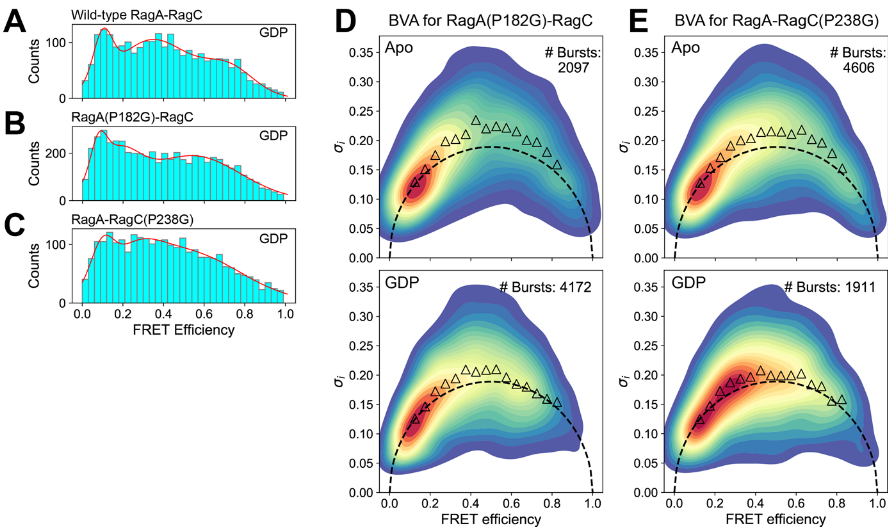

Figure S6. Global conformation of the hinge mutants.

A-C. FRET histograms of wild-type RagA-RagC (A), RagA(P182G)-RagC (B), and RagA-RagC(P238G) (C) in the dual GDP-loaded state.

D. BVA of RagA(P182G)-RagC in the apo (top panel) and GDP-loaded state (bottom panel).

E. BVA of RagA-RagC(P238G) in the apo (top panel) and GDP-loaded state (bottom panel).
